# Role of hyperhomocysteinemia in atherosclerosis: from bench to bedside

**DOI:** 10.1080/07853890.2025.2457527

**Published:** 2025-02-03

**Authors:** Wende Tian, Jianqing Ju, Baoyi Guan, Tongxin Wang, Jiqian Zhang, Luxia Song, Hao Xu

**Affiliations:** a National Clinical Research Center for Chinese Medicine Cardiology, Xiyuan Hospital, China Academy of Chinese Medical Sciences, Beijing China;; b Graduate School, China Academy of Chinese Medical Sciences, Beijing China;; c Department of Internal Medicine-Cardiovascular, The First Affiliated Hospital of Guangzhou University of Chinese Medicine, Guangzhou, China; d Graduate School, Beijing University of Chinese Medicine, Beijing, China

**Keywords:** Hyperhomocysteinemia, atherosclerosis, inflammation, oxidative stress, epigenetics, lipoprotein metabolism

## Abstract

**Background:**

Atherosclerosis is a leading cause of global mortality, driven by complex interactions between genetic, metabolic, and environmental factors. Among these, hyperhomocysteinemia (HHcy) has emerged as a significant and modifiable risk factor, contributing to endothelial dysfunction, oxidative stress, and vascular inflammation. Despite increasing recognition of its role in atherogenesis, the precise mechanisms and clinical implications of HHcy remain incompletely understood, necessitating a comprehensive review to connect recent mechanistic insights with practical applications.

**Methods:**

We analyzed the various mechanisms whereby HHcy accelerates the progression of atherosclerosis, and conducted a comprehensive review of publications in the fields of HHcy and atherosclerosis.

**Results:**

HHcy promotes atherosclerosis through several mechanisms, including inflammation, oxidative stress, epigenetic modification, and lipoprotein metabolism alteration. Moreover, this discussion extends to current strategies for the prevention and clinical management of HHcy-induced atherosclerosis.

**Conclusion:**

This review consolidates and elucidates the latest advancements and insights into the role of HHcy in atherosclerosis. The comprehensive narrative connects fundamental research with clinical applications. Contemporary studies highlight the complex interplay between HHcy and atherosclerosis, establishing HHcy as not only a contributing risk factor but also an accelerator of various atherogenic processes.

## 1. Introduction

Atherosclerosis, a chronic inflammatory disorder primarily affecting large arteries, is frequently associated with complications such as plaque rupture and thrombosis, which can trigger severe clinical events. It remains a leading global cause of morbidity and mortality, contributing significantly to the prevalence of cardiovascular and cerebrovascular conditions, including stroke and coronary artery disease [1–3].

The etiology of atherosclerosis is multifactorial, involving dyslipidemia, hypertension, smoking, diabetes, obesity, and unhealthy lifestyle choices [4]. Among these risk factors, hyperhomocysteinemia (HHcy), defined as fasting plasma homocysteine levels exceeding 15 μM, has emerged as an autonomous contributor to atherogenesis [5,6]. The association between elevated total homocysteine (tHcy) levels and stroke prevention was first recognized in 1962 with the identification of homocystinuria, a rare metabolic disorder caused by homozygous cystathionine beta-synthase deficiency. This condition, characterized by markedly elevated tHcy levels, manifests clinically as cognitive impairment, seizures, ectopia lentis, and childhood-onset strokes [7,8].

In 1969, McCully KS provided pivotal evidence linking vascular lesions to moderate and severe HHcy caused by inherited defects in one-carbon metabolism. This research suggested that even mild elevations in tHcy could accelerate atherosclerosis progression [9]. Following this groundbreaking work, interest in the relationship between elevated tHcy levels and heightened risk of atherosclerosis and stroke expanded rapidly.

Subsequent experimental studies using cellular and animal models have elucidated multiple mechanisms by which HHcy promotes atherogenesis. These include the inhibition of endothelial cell proliferation, suppression of high-density lipoprotein (HDL) synthesis, promotion of smooth muscle cell growth, and increased oxidation of low-density lipoprotein (LDL). Furthermore, HHcy has been shown to enhance the production of chemokines and reactive oxygen species (ROS), which can destabilize atherosclerotic plaques, increasing their susceptibility to rupture and subsequent occlusion.

The prevalence of HHcy varies significantly across regions. A meta-analysis of 29 studies involving 338,660 participants found that the prevalence of HHcy in China rose to 37.2% between 2013 and 2018, up from 27.5% in the previous two decades [10]. This rate is higher than in the U.S. (6.9%) [11] and Canada (19.1%) [12] but lower than that in Iran (73.1%) [13] and parts of Africa (62.3%) [14]. In the U.S., 1.8% of the population tested for homocysteine had levels above 30 μmol/L, with an estimated 33,000 individuals potentially undiagnosed due to limited screening [15].

Elevated plasma homocysteine (Hcy) is widely acknowledged as an independent risk factor for cardiovascular disease (CVD) [16–19]. The association between increased Hcy levels and atherosclerosis-related conditions, including carotid plaque formation, coronary artery calcification (CAC), and peripheral arterial disease (PAD), is well documented. A meta-analysis of 26 studies revealed that each 5 μmol/L rise in Hcy levels results in a 20% increase in the risk of coronary heart disease (CHD), irrespective of common risk factors such as hypertension, smoking, and diabetes [20]. Notably, elevated Hcy levels account for approximately 10% of coronary artery disease (CAD) cases in the population, with a strong dose-dependent positive relationship between plasma Hcy concentrations and CVD risk [21].

A Chinese study involving 474 participants found that individuals with Hcy concentrations of 12.56 μmol/L or higher had a 2.28-fold greater likelihood of developing carotid plaques over a period of 6.8 years [22]. In a similar study with 1,441 women suffering from acute coronary syndrome (ACS), the cumulative incidence of major adverse cardiovascular events (MACE) over five years was 12.6% for those in the highest Hcy quartile (14.1–64.0 μmol/L), with elevated Hcy levels serving as an independent predictor of future cardiovascular complications [23].

Further research has confirmed that individuals with Hcy concentrations ≥ 10 μmol/L are at more than double the risk of developing PAD [18,24]. The Multi-Ethnic Study of Atherosclerosis (MESA) demonstrated that Hcy levels exceeding 12 μmol/L were associated with over twice the risk of CAC and descending thoracic aortic calcification (DTAC), even after accounting for other cardiovascular risk factors [25].

Moreover, elevated Hcy levels have been linked to worse short-term outcomes for acute myocardial infarction patients undergoing percutaneous coronary intervention (PCI), including higher rates of heart failure, cardiac rupture, and death [26].

These findings underscore HHcy as a modifiable risk factor for the onset and progression of atherosclerosis, which significantly increases cardiovascular risk across diverse populations, with broad implications for both prevention and treatment strategies. Given the widespread occurrence of HHcy and its contribution to atherosclerosis, this review synthesizes the latest research on the role of HHcy in atherosclerosis, from fundamental mechanisms to clinical applications, providing insights into potential therapeutic approaches and highlighting areas for future investigation.

## 2. Hcy biosynthetic and metabolic pathway

Hcy, a non-proteinogenic amino acid containing a sulfhydryl group, serves as a crucial intermediary in the typical metabolic processes of methionine in mammals [27]. Butz and de Vigneaud first described Hcy in 1932, when they identified homologs of cysteine and cystine, which were subsequently designated as Hcy and homocystine. These compounds were characterized as possessing the structural attributes of a sulfhydryl amino acid [28,29].

Humans can only synthesize Hcy through the transmethylation of the essential amino acid methionine, which is obtained from the diet. Methionine undergoes ATP-dependent activation and is subsequently converted into S-adenosylmethionine (SAM) *via* a reaction facilitated by SAM synthetase, which is sometimes referred to as methionine adenosyltransferase (MAT). Subsequently, SAM undergoes a particular methyltransferase (MT)-mediated process, leading to its conversion into S-adenosylhomocysteine (SAH), which then undergoes rapid hydrolysis *via* SAH hydrolase, resulting in the formation of Hcy and adenosine [30,31].

**Figure 1.**
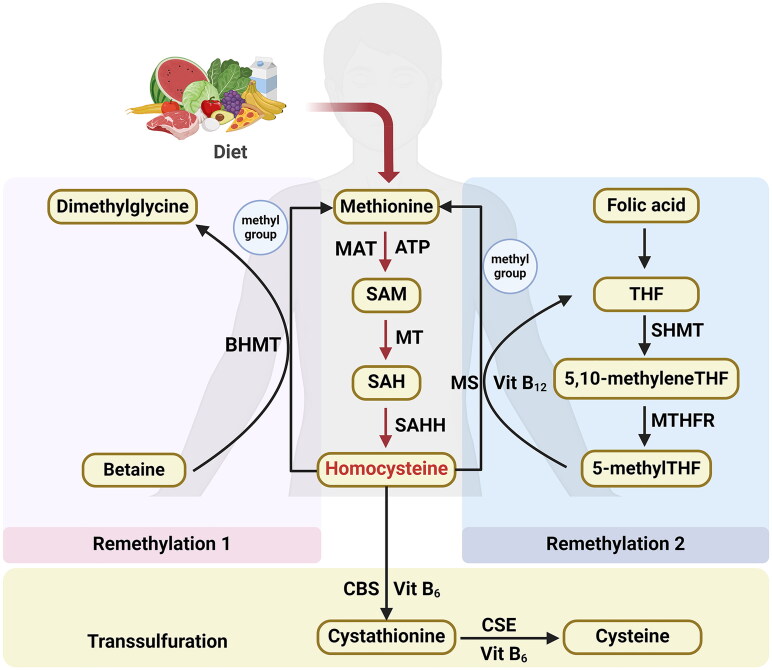
Concise pathway for homocysteine biosynthesis and metabolism. Abbreviations: BHMT: Betaine-homocysteine S-methyltransferase, MAT: Methionine adenosyltransferase, SAM: S-adenosylmethionine, MT: Methyltransferase, SAH: S-adenosylhomocysteine; SAHH: S-adenosylhomocysteine; THF: Hydrolase tetrahydrofolate, SHMT: Serine hydroxymethyltransferase, MS: Methionine synthase, 5-methylTHF: N-5-methyltetrahydrofolate, MTHFR: 5:10-methylenetetrahydrofolate reductase, CBS: Cystathionine β-synthase, CSE: Cystathionine γ-lyase, vit: Vitamin. *This figure was created using Biorender.com.*

The metabolism of Hcy is governed by two distinct pathways: remethylation and transsulfuration. Within the remethylation route, Hcy is transformed into methionine by gaining a methyl group. This essential methyl group is derived from betaine in the liver and kidneys (denoted as Remethylation 1) or from N-5-methyltetrahydrofolate (5-methylTHF) across various tissues (referred to as Remethylation 2). Remethylation 1 is characterized by the action of betaine-Hcy S-methyltransferase (BHMT) 2, a cytosolic enzyme that relies on zinc. BHMT mediates the donation of a methyl group from betaine to Hcy, culminating in the production of dimethylglycine and methionine [32]. Remethylation 2 intricately connects the metabolism of Hcy to the internal dynamics of the folate cycle within cells [33]. The produced tetrahydrofolate (THF) is further transformed into 5,10-methylene THF *via* a catalytic process facilitated by serine hydroxymethyltransferase (SHMT). Afterward, 5,10-methylene THF is further refined into 5-methylTHF *via* the action of 5,10-methylenetetrahydrofolate reductase (MTHFR) [34,35]. With vitamin B12 acting as a cofactor, methionine synthase (MS) catalyzes the transfer of a methyl group from 5-methylTHF to Hcy, thereby producing methionine and regenerating THF [36]. During the transsulfuration pathway, Hcy is further metabolized to cysteine. This transformation begins with the enzyme cystathionine β-synthase (CBS), which catalyzes the formation of cystathionine from Hcy. Subsequently, cystathionine γ-lyase (CSE) converts cystathionine into the amino acid cysteine. The entire biochemical pathway relies on the presence of vitamin B6. Figure 1 presents a detailed schematic illustrating the pathways of Hcy biosynthesis and metabolism.

Although there is no definitive cutoff value for normal plasma Hcy levels, total Hcy levels below 15 µmol/L are generally considered normal [37]. Ashjazadeh et al. [38] conducted a case–control study of 171 stroke patients and 86 matched controls, revealing that the mean fasting Hcy levels were significantly higher in stroke patients (16.2 μmol/L) than in controls (13.5 μmol/L). Elevated Hcy levels were particularly associated with the cardioembolic stroke subtype, with an adjusted odds ratio of 2.17 for Hcy levels above 15 μmol/L. Similarly, Nygård et al. [39] conducted a prospective study of 587 patients with CAD and found that elevated Hcy levels (≥ 15 μmol/L) were strongly associated with increased mortality over a median follow-up of 4.6 years compared to levels below 9 μmol/L.

HHcy, which refers to elevated Hcy levels, is classified into three categories: mild (15–30 µmol/L), intermediate (30–100 µmol/L), and severe (> 100 µmol/L). Severe cases are rare and usually result from genetic mutations affecting Hcy metabolism, such as defects in enzymes such as CBS, methionine synthase (MS), and 5,10-methylenetetrahydrofolate reductase (MTHFR) [40]. For example, MTHFR polymorphisms are associated with mild to moderate HHcy owing to reduced enzyme activity, whereas CBS deficiencies can dramatically increase Hcy levels in severe cases.

Environmental factors, including deficiencies in folic acid and vitamins B6 and B12, are more likely to play a role in mild to moderate HHcy. Folic acid (as 5-methylTHF) acts as a methyl donor in the conversion of Hcy into methionine, an MS-catalyzed reaction that requires vitamin B12 as a co-factor [41]. Vitamin B6 is essential for CBS, as it converts Hcy into cystathionine and subsequently cysteine. Deficiencies of these vitamins lead to elevated Hcy levels.

Other factors contributing to HHcy include physiological factors (e.g. aging, male sex, menopause), lifestyle choices (e.g. smoking, coffee consumption), chronic diseases (e.g. kidney failure, liver disease, diabetes, hypothyroidism), and certain medications (e.g. methotrexate, estrogen-based contraceptives) that impair Hcy metabolism [42–44].

Moderate HHcy (15–30 µmol/L) is often caused by poor diet, mild folate, B12, or B6 deficiencies, heterozygosity for CBS defects, hypothyroidism, impaired renal function, or intake of certain medications [37]. Intermediate HHcy (30–100 µmol/L) is typically due to more severe B12 or folate deficiencies or renal failure [37]. Severe HHcy (> 100 µmol/L) is usually caused by severe B12 deficiency or genetic conditions such as homocystinuria [45].

## 3. Summary of the pathological impact of HHcy on blood vessels

### 3.1. Prothrombotic effects of HHcy

HHcy induces a prothrombotic state through several interconnected mechanisms, disrupting the hemostatic balance within the vascular endothelium. Elevated Hcy levels contribute to a procoagulant environment by stimulating platelet production of thromboxane A2, a potent promoter of platelet aggregation and clot formation [46]. Furthermore, Hcy interferes with anticoagulant pathways by activating factor V and impairing the protein C system through downregulation of thrombomodulin expression, resulting in reduced synthesis and activity of critical anticoagulant proteins [47].

Hcy further intensifies coagulation by upregulating tissue factor (TF), a primary initiator of the extrinsic coagulation cascade [48]. Hcy enhances both the expression and activity of TF in endothelial cells, which correlates with increased plasma levels of TF and factor VII, particularly in patients with CAD, thereby increasing thrombotic risk [49,50]. Additionally, Hcy impairs fibrinolysis by raising levels of plasminogen activator inhibitor-1 (PAI-1) and disrupting the interaction between fibrin and tissue plasminogen activator (tPA), thereby extending clot stability and elevating thrombosis risk [51,52]. Furthermore, N-homocysteinylation of fibrinogen, especially in individuals with cystathionine β-synthase deficiency, contributes to fibrinolytic dysfunction [53]. Hcy-thiolactone and its derivatives are also associated with CVDs and serve as independent predictors of acute myocardial infarction [54,55].

Enhanced platelet reactivity increases the prothrombotic potential of HHcy. Increased production of bioactive lipids, such as thromboxane A2 and 8-iso-prostaglandin F2α, has been observed in patients with homocystinuria, promoting further platelet aggregation and thrombin formation [56]. This effect is evident in both animal models and human studies involving methionine loading [56,57]. Collectively, these mechanisms underscore the pivotal role of Hcy in thrombosis and highlight its broader implications in the progression of CVDs.

### 3.2. Impact of HHcy on endothelial dysfunction

HHcy plays a critical role in endothelial dysfunction, primarily by compromising vascular integrity through a complex interplay of biochemical processes. Elevated Hcy levels trigger several damaging mechanisms in endothelial cells (ECs), which are predominantly driven by oxidative stress, inflammation, and disruptions in nitric oxide (NO) signaling. Collectively, these pathways accelerate atherosclerosis progression and intensify endothelial injury.

A key mechanism whereby Hcy contributes to endothelial dysfunction is the overproduction of ROS. This occurs primarily through the activation of NADPH oxidase, mitochondrial dysfunction, and xanthine oxidase [58]. Excessive ROS generation overwhelms endothelial antioxidant defense systems, creating a feedback loop with endoplasmic reticulum (ER) stress, which further intensifies cellular damage [59]. A hallmark of ER stress is the accumulation of misfolded or unfolded proteins within the ER, which intensifies EC dysfunction and leads to apoptosis, pyroptosis, and ferroptosis [60–63]. Pyroptosis, which is mediated by caspase-1-dependent inflammasome activation and gasdermin proteins, amplifies local and systemic inflammation, accelerating atherosclerosis by releasing inflammatory cytokines such as interleukin (IL)-1β and IL-18 [63].

Moreover, HHcy significantly impairs NO bioavailability, which is a crucial factor in maintaining vascular homeostasis [64]. Elevated Hcy levels reduce NO synthesis through multiple mechanisms, including the accumulation of asymmetric dimethylarginine (ADMA), an endogenous inhibitor of nitric oxide synthase (NOS). By inhibiting dimethylarginine dimethylaminohydrolase (DDAH), the enzyme responsible for degrading ADMA, Hcy leads to ADMA accumulation, which directly inhibits NOS and diminishes NO production [59,65]. Additionally, Hcy activates the protein kinase C (PKC) pathway, further disrupting NO synthesis. The resulting reduction in NO weakens its vasodilatory, anti-inflammatory, and antithrombotic properties, promoting endothelial injury, vasoconstriction, and thrombosis [58,66,67].

The interplay between oxidative stress and the ER stress creates a vicious cycle that further compromises endothelial function. Hcy-induced ER stress, characterized by the upregulation of ER oxidoreductin-1α (Ero-1α) and downregulation of glutathione peroxidase 7 (GPX7), disrupts ER homeostasis, leading to apoptosis and the release of pro-inflammatory mediators [68].

Inflammation plays a central role in HHcy-induced endothelial dysfunction. Hcy activates the nuclear factor-kappa B (NF-κB) pathway, triggering the expression of pro-inflammatory cytokines, including IL-1β, IL-6, IL-8, and tumor necrosis factor-alpha (TNF-α) [69–72]. These cytokines are instrumental in recruiting circulating monocytes to areas of endothelial damage where they differentiate into pro-inflammatory macrophages [73]. These macrophages eventually transform into foam cells, contributing to the formation of atherosclerotic plaques. In addition to promoting inflammation, Hcy upregulates PAI-1 and induces EC senescence, with telomere shortening implicated in this process [74]. Senescent ECs adopt a pro-inflammatory phenotype known as the senescence-associated secretory phenotype, which further aggravates endothelial injury and inflammation.

Disruption of the hydrogen sulfide (H_2_S) signaling pathway, another key regulator of vascular homeostasis, significantly contributes to HHcy-induced endothelial dysfunction. H_2_S, produced *via* the transsulfuration pathway by enzymes such as CBS and CSE, plays a protective role by mitigating oxidative stress, promoting EC migration, and enhancing angiogenesis [75,76]. In HHcy, reduced expression of CBS and CSE results in decreased H_2_S production, impairing endothelial repair mechanisms and worsening endothelial dysfunction [59]. The loss of the protective effects of H_2_S against Hcy-induced damage is notable, as exogenous H_2_S mitigates mitochondrial toxicity and restore endothelial function [76]. Furthermore, the interaction between H_2_S and NO is of particular importance, as H_2_S can enhance NO production by promoting NOS phosphorylation, indicating its synergistic role in vascular protection [77].

HHcy also disrupts cellular methylation by accumulating SAH, an inhibitor of methyltransferases. Elevated SAH creates a hypomethylating environment that impairs the methylation of DNA, RNA, and proteins. This epigenetic dysregulation upregulates adhesion molecules such as intercellular adhesion molecule-1 (ICAM-1) and vascular cell adhesion molecule-1 (VCAM-1), promoting monocyte adhesion and endothelial inflammation [78]. Additionally, Hcy-induced DNA hypomethylation impairs endothelial progenitor cell proliferation, further compromising endothelial repair [79].

Protein homocysteinylation, a post-translational modification in which Hcy thiolactone binds to lysine residues on proteins, adds another layer of damage. This modification alters the function of key proteins, such as HDL and angiotensin-converting enzyme (ACE) [80]. Homocysteinylated HDL loses its protective ability, whereas homocysteinylated ACE enhances angiotensin II activity, increasing oxidative stress and worsening endothelial injury [81]. Additionally, impaired protein quality control mechanisms, including the unfolded protein response (UPR) and autophagy, further intensify endothelial dysfunction [82].

In conclusion, HHcy induces endothelial dysfunction through a multifactorial process involving oxidative stress, inflammation, impaired NO signaling, H_2_S pathway disruption, epigenetic dysregulation, and protein homocysteinylation. These interconnected mechanisms collectively drive the development and progression of atherosclerosis, emphasizing the need for targeted therapeutic strategies to mitigate Hcy-induced vascular damage.

### 3.3. Impact of HHcy on the medial layer of large arteries and its role in vascular remodeling

The medial layer of large arteries, composed of smooth muscle cells (SMCs), elastin, collagen, and proteoglycans, is crucial to maintaining vascular integrity and function. This layer provides the mechanical strength and elasticity required for proper vascular function. Disruptions in its structure and function can lead to pathological vascular remodeling and contribute to CVDs such as atherosclerosis, hypertension, and aneurysms. HHcy disrupts the medial layer, inducing significant structural and biochemical changes.

Under normal physiological conditions, SMCs in the medial layer maintain a contractile phenotype, ensuring vascular tone and stability. However, in HHcy, SMCs undergo a phenotypic shift from contractile to synthetic, characterized by increased SMC proliferation, collagen synthesis, and extracellular matrix (ECM) deposition [83]. This synthetic phenotype mirrors the pathological changes observed in atherosclerosis, in which excessive SMC proliferation leads to arterial thickening and increased vascular stiffness. Studies using various animal models of HHcy, including CBS knockout mice and Hcy-supplemented rats, have shown that HHcy induces collagen deposition, increased wall thickness, and arterial stiffness, predisposing the vasculature to hypertension and remodeling [84–88].

Elastin, a key ECM component that imparts elasticity to arteries, is particularly susceptible to degradation in the presence of HHcy. The activity of elastin-degrading enzymes, such as matrix metalloproteinases (MMP-2, MMP-9), neutrophil elastase, and cathepsins (K and S), increases in HHcy, leading to accelerated elastin fragmentation. This degradation compromises arterial wall integrity by reducing elasticity and contributing to vascular stiffness. Additionally, elastin fragmentation increases the susceptibility of arteries to pressure-induced damage, increasing the risk of aneurysm formation, particularly in the abdominal aorta [84,89].

HHcy also promotes the irreversible homocysteinylation of ECM proteins, including elastin and fibrillin-1, thereby impairing their function and further exacerbating vascular stiffness. This results in a decrease in elastin and an increase in collagen, leading to arterial wall thickening—an early hallmark of atherosclerosis and vascular remodeling. Moreover, HHcy upregulates methionyl-tRNA synthetase, which drives the homocysteinylation of key vascular proteins, further compromising the structural integrity of the medial layer and perpetuating arterial stiffness and remodeling [90].

In addition to structural changes, HHcy promotes inflammatory processes within the vasculature. Elevated Hcy levels enhance neutrophil–endothelial interactions, promoting neutrophil migration into the arterial wall and creating a pro-inflammatory environment [91]. This inflammation accelerates vascular disease progression, further worsening medial layer remodeling. Moreover, SMCs exposed to HHcy produce profibrotic growth factors, such as connective tissue growth factor (CTGF) and transforming growth factor β1 (TGF-β1), contributing to fibrosis and plaque formation in atherosclerotic lesions [90,92].

An important aspect of the impact of HHcy on vascular remodeling is its interaction with methyl donor deficiency (MDD), particularly during pregnancy and fetal development [90]. Deficiencies in vitamins B12 and folate, which are crucial to Hcy metabolism, intensify HHcy, leading to long-term adverse effects on vascular health. Fetal programing under MDD conditions can alter cardiovascular development in offspring, predisposing them to vascular remodeling and cardiovascular diseases later in life [90]. Indeed, animal studies have shown that offspring exposed to MDD in utero exhibit reduced elastin content, increased collagen deposition, and elastin fragmentation in the aorta, resulting in arterial stiffening and hypertension [90]. These structural changes are associated with increased expression of TGF-β1, elevated levels of α-smooth muscle actin, and enhanced protease activity, indicating a profibrotic and pro-inflammatory state in the arterial wall [93].

In summary, HHcy induces profound structural and biochemical changes in the medial layer of large arteries, driving vascular remodeling characterized by SMC proliferation, elastin degradation, collagen deposition, and inflammatory responses. These cumulative effects contribute to the development of atherosclerosis, hypertension, and aneurysms. MDD during pregnancy further amplifies these risks through fetal programming, meaning that early intervention is essential to mitigate long-term cardiovascular outcomes.

### 3.4. Impact of HHcy on adventitial inflammation and vascular remodeling

HHcy significantly affects the adventitial layer of large arteries, contributing to vascular remodeling and the development of atherosclerosis. Deficiencies in B vitamins (folate, B6, and B12), particularly when combined with a high-fat diet, disrupt lipid metabolism, leading to the accumulation of proatherogenic lipoproteins in the adventitia. This phenomenon promotes inflammation and accelerates atherosclerosis progression. This process is further worsened by an altered fatty acid profile, which is characterized by increased saturated fats and decreased monounsaturated fats [94].

HHcy enhances immune activity within the adventitia by activating B cells and CD4+ T cells through the pyruvate kinase M2 (PKM2)-cAMP response element-binding protein 1 (CREB1)-class II transactivator (CIITA) pathway, thereby amplifying inflammation [95]. In models of abdominal aortic aneurysm (AAA), HHcy exacerbates Angiotensin II (Ang II)-induced aneurysm formation and increases immune cell infiltration, along with elevated production of cytokines such as interleukin-6 (IL-6) and C-C motif chemokine ligand 2. This immune response is driven by nicotinamide adenine dinucleotide phosphate oxidase 4 (Nox4), which leads to the production of ROS, activating adventitial fibroblasts [96]. Studies have shown that folic acid supplementation and Nox4 inhibition attenuate these effects, signifying potential therapeutic interventions [96].

In the progression of AAA, HHcy activates nucleotide-binding domain leucine-rich repeat-containing protein 3 (NLRP3) inflammasome in macrophages, triggering inflammation and matrix degradation within the adventitia [97]. Mitochondrial ROS play a critical role in this pathway, and their inhibition has been shown to reduce inflammasome activity and subsequent inflammation, offering another potential therapeutic target [97].

In addition, HHcy intensifies vascular remodeling in balloon-injured rat carotid arteries by upregulating type I collagen expression in the adventitial layer. This phenomenon is mediated through the activation of the Angiotensin II type 1 receptor (AT1R) in adventitial fibroblasts, leading to excessive collagen deposition, vascular stiffness, and fibrosis. Inhibition of AT1R with valsartan has been shown to reduce these remodeling effects, highlighting the key role of AT1R signaling in HHcy-induced vascular damage [98]. Correspondingly, HHcy promotes atherosclerosis by enhancing inflammation and fibrosis in the adventitia *via* AT1R activation [99]. The protein kinase C (PKC) and extracellular signal-regulated kinase 1/2 (ERK1/2) pathways are also activated, further promoting fibroblast proliferation and migration, which drive adventitial inflammation and atherosclerosis. Telmisartan, another AT1R blocker, has been demonstrated to mitigate these effects, further highlighting the importance of targeting AT1R signaling in HHcy-induced vascular remodeling [99].

Furthermore, the deubiquitinase cylindromatosis (CYLD) plays a critical role in regulating adventitial fibroblast activation in HHcy by stabilizing Nox4, which drives ROS production and vascular remodeling. Silencing CYLD reduces fibroblast activation, inflammation, and AAA formation, highlighting CYLD as a potential therapeutic target in HHcy-related vascular diseases [100].

In summary, HHcy drives vascular remodeling and atherosclerosis through a multifaceted process involving disruption of lipid metabolism, immune cell activation, production of ROS, fibroblast activation, and upregulation of the inflammatory and fibrotic pathways. Targeting key pathways such as Nox4, AT1R, and CYLD offers promising therapeutic strategies to mitigate the adverse vascular effects of HHcy.

## 4. Mechanism of HHcy in atherosclerosis

**Figure 2.**
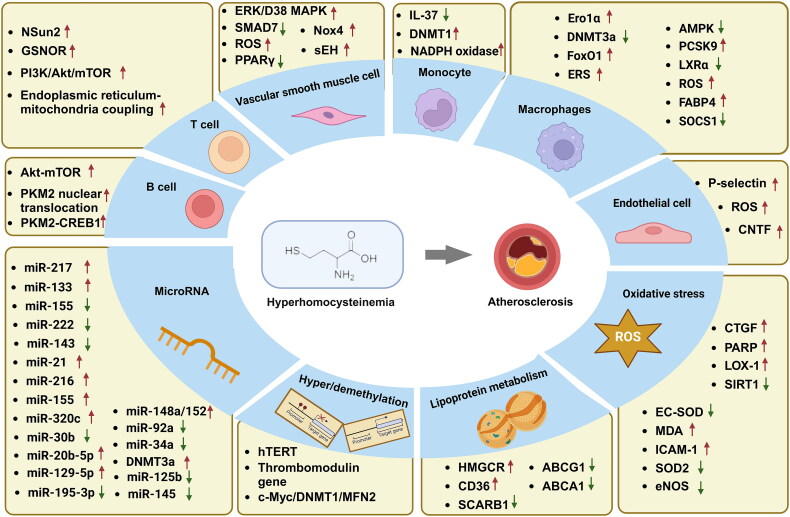
Pathophysiological mechanisms linking hyperhomocysteinemia to atherosclerosis. This figure delineates the complex pathophysiological pathways by which hyperhomocysteinemia (HHcy) drives the development and progression of atherosclerosis. The Central molecule, homocysteine (Hcy), induces numerous cellular and molecular dysfunctions across various cell types, contributing to atherosclerotic plaque formation and cardiovascular disease. In vascular smooth muscle cells, HHcy upregulates several signaling pathways, including SMAD family member 7 (SMAD7), reactive oxygen species (ROS), peroxisome proliferator-activated receptor gamma (PPARγ), NADPH oxidase 4 (Nox4), extracellular signal-regulated kinase/D38 mitogen-activated protein kinase (ERK/D38 MAPK), and soluble epoxide hydrolase (sEH), all of which promote vascular remodeling and dysfunction. In monocytes, HHcy increases levels of interleukin-37 (IL-37), DNA methyltransferase 1 (DNMT1), and NADPH oxidase, enhancing pro-inflammatory and oxidative activities. In macrophages, HHcy elevates the expression of pro-inflammatory and pro-atherogenic molecules such as endoplasmic reticulum oxidoreductin 1-alpha (Ero1α), DNA methyltransferase 3a (DNMT3a), forkhead box protein O1 (FoxO1), ROS, fatty acid-binding protein 4 (FABP4), and proprotein convertase subtilisin/kexin type 9 (PCSK9), while downregulating AMP-activated protein kinase (AMPK). This dysregulation increases lipid uptake, foam cell formation, and inflammation, exacerbating atherogenesis. In endothelial cells, HHcy upregulates P-selectin, ROS, and ciliary neurotrophic factor (CNTF), leading to endothelial dysfunction, an early hallmark of atherosclerosis. These changes promote leukocyte adhesion, oxidative stress, and impaired vascular homeostasis, accelerating plaque formation. In T cells, HHcy primarily upregulates the tRNA methyltransferase NSun2 (NOP2/sun RNA methyltransferase family member 2), which methylates interleukin-17A (IL-17A) mRNA, enhancing its expression and contributing to inflammation. Additionally, HHcy increases endoplasmic reticulum (ER)-mitochondria coupling, driving metabolic reprogramming and T cell activation. HHcy also induces the expression of pyruvate kinase M2 (PKM2) through the phosphoinositide 3-kinase (PI3K)/Akt/mammalian target of rapamycin (mTOR) pathway, crucial for metabolic shifts in T cells. Lastly, HHcy enhances the expression of S-nitrosoglutathione reductase (GSNOR), promoting T cell activation *via* protein denitrosylation. In B cells, HHcy induces the nuclear translocation of PKM2, which activates the transcription of antigen-presentation-related genes. HHcy also activates the Akt/mTOR pathway, driving metabolic reprogramming, leading to B cell activation and immune response enhancement. HHcy-induced dysregulation of microRNAs (miRNAs) plays a critical role in modulating key atherosclerosis-related pathways. Notably, upregulation is seen in miR-217, miR-133, miR-155, miR-21, miR-148a/152, miR-320c, and miR-129-5p, while miR-222, miR-143, miR-34a, miR-125b, and miR-145 are downregulated. These miRNAs regulate various processes, including inflammation, vascular smooth muscle cell proliferation, and lipid metabolism, linking HHcy to enhanced atherogenesis. HHcy also disrupts DNA methylation patterns by upregulating key genes involved in methylation, such as c-Myc, DNMT1, mitofusin-2 (MFN2), and human telomerase reverse transcriptase (hTERT), as well as the thrombomodulin gene. These epigenetic changes play a significant role in the pathogenesis of HHcy-induced atherosclerosis. Dysregulated lipid metabolism is another hallmark of HHcy, as evidenced by the upregulation of 3-hydroxy-3-methylglutaryl-CoA reductase (HMGCR), cluster of differentiation 36 (CD36), and scavenger receptor class B type 1 (SCARB1), along with the downregulation of ATP-binding cassette transporter G1 (ABCG1) and ATP-binding cassette transporter A1 (ABCA1). These changes promote lipid accumulation in arterial walls, leading to foam cell formation and plaque development. The Central role of oxidative stress in HHcy-induced atherosclerosis is highlighted by increased ROS levels and the upregulation of pro-oxidative molecules such as connective tissue growth factor (CTGF), poly(ADP-ribose) polymerase (PARP), and lectin-like oxidized low-density lipoprotein receptor-1 (LOX-1), while protective enzymes such as extracellular superoxide dismutase (EC-SOD), superoxide dismutase 2 (SOD2), and endothelial nitric oxide synthase (eNOS) are downregulated, contributing to further vascular damage and dysfunction. *This figure was created using Biorender.com.*

The mechanism by which HHcy contributes to atherosclerosis is detailed below, with a comprehensive summary provided in Figure 2.

### 4.1. HHcy and inflammation

Inflammation is an adaptive, protective response triggered by harmful stimuli such as tissue damage and infections [101]. The acute phase of inflammation is typically self-limiting, concluding in tissue repair; however, chronic inflammation occurs when the initial trigger persists or the resolution process malfunctions [102]. Prolonged, low-level inflammation has been implicated in the development of numerous diseases, including atherosclerosis. Atherosclerosis, a chronic inflammatory disease, affects medium-to-large arteries, leading to the accumulation of fatty and fibrous deposits within arterial walls [103].

Inflammation is central to atherogenesis, from the initial activation of ECs by modified lipids to the eventual rupture of atherosclerotic plaques. The progression of atherosclerosis is driven by a complex interplay between innate and adaptive immune responses, involving immune cells such as monocytes, macrophages, neutrophils, T cells, and B cells [104].

HHcy intensifies atherosclerosis progression by amplifying inflammatory processes [105]. Studies have shown significant correlations between elevated Hcy levels and increased concentrations of inflammatory markers, such as complement component 4 (C4), C-reactive protein (CRP), and immunoglobulin M (IgM). Hcy is inversely related to C4 but positively associated with CRP and IgM, suggesting that HHcy promotes immune activation and inflammation, potentially contributing to cardiovascular diseases [106]. In patients with acute myocardial infarction, elevated Hcy levels correlate with increased total cholesterol, low-density lipoprotein cholesterol (LDL-C), CRP, and pro-inflammatory cytokines, including TNF-α and IL-6, while showing an inverse correlation with the anti-inflammatory cytokine IL-10 [107]. Elevated Hcy, which has emerged as an independent risk factor for MACE, may serve as a biomarker for predicting cardiovascular outcomes [107].

The relationship between HHcy, vitamin D deficiency, and inflammation in atherosclerotic plaques in patients with CAD underscores the role of HHcy in vascular inflammation [108]. A positive correlation between Hcy levels and plaque inflammation has been observed, whereas vitamin D levels are negatively correlated with the inflammatory response [108]. Patients with elevated Hcy and low vitamin D levels exhibit heightened inflammatory responses, marked by increased neutrophil infiltration and atherosclerotic plaque calcification, suggesting that both HHcy and vitamin D deficiency accelerate atherosclerosis [108].

Hypersensitive C-reactive protein (hs-CRP), a critical marker of inflammation, plays a significant role in atherosclerosis intensified by HHcy [30]. Elevated levels of both Hcy and hs-CRP are associated with greater CHD severity, particularly in patients with acute myocardial infarction or unstable angina pectoris (UAP), further highlighting their roles as predictors of disease severity [109].

Studies show that HHcy correlates with increased carotid intima-media thickness, unstable plaques, and intracranial and extracranial arteries [105]. HHcy promotes atherosclerosis by driving inflammation, as evidenced by elevated hs-CRP levels, which may lead to lacunar infarction [105]. Zhang et al^.^ [110] further explored the role of Hcy in promoting carotid atherosclerotic plaque (CAP) formation *via* both inflammatory and non-inflammatory mechanisms. Their findings demonstrated that Hcy levels above 19.7 mmol/L significantly increased the risk of CAP formation, with a strong correlation between Hcy and elevated hs-CRP levels, suggesting that Hcy has a role in inflammation-driven atherosclerosis [110]. However, in the early stages of atherosclerotic thickening of the carotid artery, no independent effect of Hcy was observed, indicating that the influence of Hcy is more pronounced during plaque development [110].

Elevated Hcy levels have also been strongly associated with lacunar infarction and severe white matter lesions (WMLs), with higher Hcy concentrations correlating with greater WML severity, both of which are key manifestations of cerebral small vessel disease. Higher Hcy levels are correlated with increased severity of WMLs, as assessed by the Fazekas scale [111]. Additionally, HHcy has been shown to be linked to higher levels of hs-CRP, suggesting an inflammatory pathway [111]. These findings indicate that HHcy is a risk factor for ischemic cerebral small vessel disease and may contribute to its development through inflammatory mechanisms. Elevated levels of Hcy and CRP are also linked to worse neurological outcomes, as measured by the National Institutes of Health Stroke Scale (NIHSS^)^ [112]. Hcy has been implicated in endothelial dysfunction and plaque formation, whereas CRP contributes to vascular inflammation and thrombosis [112]. This suggests that Hcy and CRP are useful biomarkers for assessing stroke severity and predicting patient prognosis after stent treatment.

Another study examined the relationship between serum Hcy levels, inflammation, and carotid plaque stability in patients with H-type hypertension and carotid atherosclerosis [113]. Elevated Hcy levels were positively correlated with markers of inflammation, including human cartilage glycoprotein-39 (HCGP-39), TNF-α, hs-CRP, IL-1β, pentraxin 3 (PTX3), and lipoprotein-associated phospholipase A2 (Lp-PLA2), whereas they were inversely correlated with bilirubin levels (total, direct, and indirect) [113]. These inflammatory markers are linked to plaque instability, suggesting that HHcy accelerates atherosclerosis and increases the risk of plaque rupture in patients with H-type hypertension. Furthermore, a study investigating the relationship between Hcy levels, inflammation, and cerebral infarction (CI) risk in hypertensive patients revealed that patients with CI exhibited significantly higher levels of Hcy, TNF-α, IL-6, and CRP levels than non-stroke hypertensive patients and healthy controls [114]. Following treatment, reductions in Hcy, TNF-α, and NIHSS scores were observed, suggesting a positive correlation between elevated Hcy and inflammatory markers and the incidence of CI in hypertension [114].

**Figure 3.**
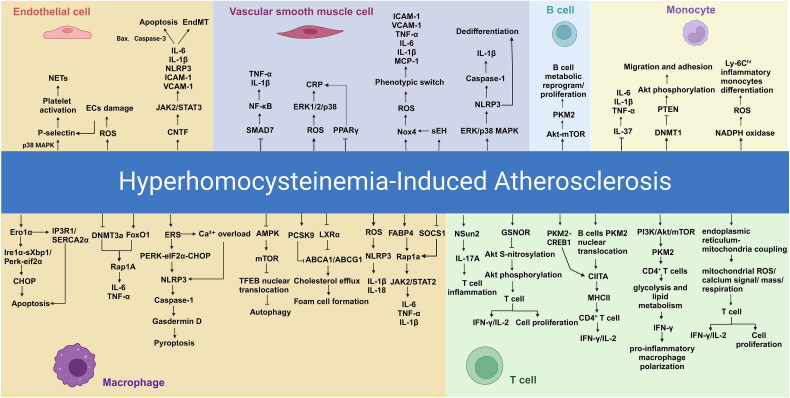
Inflammatory mechanisms of hyperhomocysteinemia (HHcy) in atherosclerosis. This figure illustrates the cellular and molecular pathways by which hyperhomocysteinemia (HHcy) drives inflammation and contributes to atherosclerosis. In endothelial cells (ECs), HHcy promotes oxidative stress through reactive oxygen species (ROS) generation, leading to EC damage, platelet activation *via* P-selectin, and neutrophil extracellular trap (NET) formation. HHcy triggers the expression of inflammatory cytokines, such as interleukin-6 (IL-6), interleukin-1β (IL-1β), and nucleotide-binding oligomerization domain-like receptor family pyrin domain-containing 3 (NLRP3), as well as adhesion molecules including intercellular adhesion molecule-1 (ICAM-1) and vascular cell adhesion molecule-1 (VCAM-1), leading to EC apoptosis through the bcl-2-associated X protein (Bax) and cleaved caspase-3 pathways and endothelial-to-mesenchymal transition (EndMT). Vascular smooth muscle cells (VSMCs) experience a phenotypic switch and inflammation *via* the activation of nuclear factor kappa-light-chain-enhancer of activated B cells (NF-κB) and ROS pathways, leading to the upregulation of cytokines such as tumor necrosis factor-alpha (TNF-α) and IL-1β. HHcy-induced macrophages undergo phenotypic changes and activate inflammasomes, leading to pyroptosis and exacerbation of plaque instability. Macrophage dysfunction is further driven by endoplasmic reticulum (ER) stress and autophagy disruption *via* the AMP-activated protein kinase (AMPK)-mechanistic target of rapamycin (mTOR)-transcription factor EB (TFEB) pathway, contributing to foam cell formation and cholesterol accumulation. Monocytes exhibit increased migration, adhesion, and differentiation into Ly-6C^hi^ inflammatory monocytes *via* phosphatase and tensin homolog (PTEN) suppression and protein kinase B (Akt) phosphorylation, whereas enhanced ROS production *via* nicotinamide adenine dinucleotide phosphate (NADPH) oxidase accelerates inflammation. In B cells, HHcy induces metabolic reprogramming, enhancing B cell proliferation *via* the pyruvate kinase muscle isozyme M2 (PKM2)-cAMP response element-binding protein 1 (CREB1) axis, and promoting antigen presentation and pro-inflammatory interactions with CD4^+^ T cells. T cells undergo activation through enhanced glycolysis and mitochondrial ROS production, with upregulation of interleukin-17A (IL-17A) and interferon-gamma (IFN-γ) *via* the Akt-mTOR pathway. These interactions, which involve ECs, VSMCs, macrophages, monocytes, B cells, and T cells, underscore the systemic inflammatory impact of HHcy in promoting atherosclerotic progression through various interconnected mechanisms. *This figure was created using Biorender.com.*

Although the detailed mechanisms linking these biomarkers to atherosclerosis and their interrelations are yet to be fully decoded, the prevailing data imply that inflammation may serve as a factor connecting HHcy and atherosclerosis (Figure 3).

Elevated Hcy levels have been shown to stimulate the expression of the ciliary neurotrophic factor (CNTF) in human umbilical vein endothelial cells (HUVECs), which subsequently activates the JAK2/STAT3 signaling pathway [115]. This leads to increased production of pro-inflammatory cytokines, such as IL-6, IL-1β, NLRP3, and adhesion molecules such as ICAM-1 and VCAM-1 [115]. The resulting inflammation promotes EC apoptosis *via* the Bax and cleaved caspase-3 pathways and induces the endothelial-to-mesenchymal transition (EndMT), which is characterized by elevated alpha smooth muscle actin (α-SMA) and reduced CD31 expression. MAFK is a transcription factor that enhances CNTF expression, further amplifying the inflammatory response and endothelial damage [115]. Knockdown of CNTF mitigates these effects by reducing inflammation and apoptosis, suggesting that targeting the CNTF-MAFK axis could serve as a potential therapeutic strategy for preventing endothelial dysfunction and CVDs associated with HHcy [115]. In addition, Wei et al. [116] reported that Hcy induces vascular inflammation by promoting hypermethylation of the SMAD7 promoter in human umbilical vein smooth muscle cells (HUVSMCs). This hypermethylation suppresses SMAD7 expression, which in turn upregulates the pro-inflammatory cytokines TNF-α and IL-1β *via* activation of the NF-κB signaling pathway [116]. These findings identify SMAD7 hypermethylation as a novel mechanism by which Hcy drives vascular inflammation and contributes to the development of atherosclerosis.

Macrophages, a crucial subset of immune cells, play pivotal roles in innate immunity, inflammation, wound healing, and pathogen defense [117]. During the early stages of atherosclerosis, macrophages attempt to clear excess lipids but soon transform into foam cells as lipid accumulation overwhelms this process [118]. Incomplete foam cell apoptosis leads to the formation of a necrotic core within atherosclerotic plaques composed of cholesterol and cellular debris [117]. Consequently, macrophages become central to the inflammatory dynamics of atherosclerosis, dominating immune cell populations within atherosclerotic lesions [36].

HHcy affects macrophages at both phenotypic and functional levels through multiple mechanisms. Elevated Hcy levels promote a pro-atherosclerotic phenotype in human monocytes by suppressing PTEN activity [119]. Hcy induces PTEN methylation, reducing its expression and enhancing Akt phosphorylation at serine 473, which increases monocyte migration and adhesion to inflamed ECs [119]. Inhibition of DNA methyltransferase 1 (DNMT1) or supplementation with vitamin B12 and folic acid reverses these effects, restoring PTEN function and normalizing monocyte behavior [119]. Hcy also drives the differentiation of Ly-6C^hi^ inflammatory monocytes and increases ROS production through NADPH oxidase activation [120]. Probucol, an antioxidant, reduces both Hcy-induced monocyte differentiation and ROS production by inhibiting NADPH oxidase, suggesting that its anti-atherosclerotic effects stem from oxidative stress mitigation [120].

Furthermore, HHcy exacerbates plaque instability by inducing ER stress and macrophage apoptosis [121]. Under HHcy conditions, macrophages exhibit upregulated expression of Ero1α, leading to activation of the PERK-eIF2α and IRE1α-sXBP1 pathways, which promote ER stress [121]. Knockdown of Ero1α reduces macrophage apoptosis and plaque vulnerability, whereas overexpression intensifies these effects [121]. Ero1α also modulates calcium homeostasis *via* IP3R1 and SERCA2α, further contributing to apoptosis [121]. Targeting Ero1α may represent a therapeutic strategy to mitigate HHcy-induced atherosclerosis.

Hcy also accelerates inflammation in macrophages through the Ras-associated protein 1 A (Rap1a) pathway [122]. Hcy promotes Rap1A expression *via* DNMT3a-mediated hypomethylation of the Rap1a promoter, whereas FoxO1 directly binds to the Rap1a promoter, further enhancing its expression [122]. This synergistic interaction amplifies macrophage inflammation and increases pro-inflammatory cytokines such as IL-6 and TNF-α [122]. Additionally, Xu et al. [123] demonstrated that fatty acid-binding protein 4 (FABP4) intensifies Hcy-induced macrophage inflammation by activating the JAK2/STAT2 pathway *via* Rap1a. Inhibition of SOCS1, a negative regulator of this pathway, further amplifies the inflammatory response, positioning FABP4 as a key mediator of Hcy-induced inflammation [123].

Furthermore, HHcy activates the NLRP3 inflammasome *via* a ROS-dependent mechanism. In ApoE^−/−^ mice fed a high-fat, high-methionine diet to induce HHcy, increased expression of IL-1β and IL-18 was observed, leading to macrophage infiltration into atherosclerotic lesions [124]. Silencing NLRP3 or using the antioxidant N-acetyl-L-cysteine (NAC) reduced inflammasome activation and atherosclerosis, indicating that NLRP3 activation is a key mediator of HHcy-induced vascular inflammation [124]. HHcy also promotes plaque progression by inducing macrophage pyroptosis, driven by ER stress and calcium dysregulation. Hcy disrupts ER-mitochondrial coupling, leading to Ca^2+^ overload, mitochondrial dysfunction, and increased ROS production, all of which intensify macrophage pyroptosis and plaque development [125]. Inhibiting ER stress and calcium signaling significantly reduces pyroptosis and slows plaque progression, highlighting potential therapeutic targets [125].

Hcy also impairs autophagy in macrophages by disrupting the AMPK-mTOR-TFEB signaling pathway [126]. Hcy-treated macrophages exhibit reduced levels of autophagy markers, such as LC3 and Beclin-1, and increased levels of p62, indicating autophagy inhibition [126]. Mechanistically, Hcy increases mTOR phosphorylation (inhibiting autophagy), while decreasing AMPK phosphorylation (promoting autophagy) and blocks TFEB nuclear translocation, further impairing autophagy [126]. The suppression of autophagy may intensify atherosclerosis by impairing macrophage function.

Finally, Hcy accelerates atherosclerosis by inhibiting liver X receptor alpha (LXRα)-mediated cholesterol efflux in macrophages. Specifically, Hcy downregulates the ATP-binding cassette transporters A1 (ABCA1) and G1 (ABCG1), which are responsible for transporting cholesterol from macrophages to the extracellular acceptors apoA-I and HDL, respectively. This inhibition of cholesterol transport leads to increased lipid accumulation and foam cell formation [127]. Activation of LXRα with the agonist T0901317 restores ABCA1 and ABCG1 levels, promotes cholesterol efflux, and alleviates Hcy-induced lipid accumulation, thereby reducing atherosclerotic lesion size [127]. Additionally, HHcy upregulates the proprotein convertase subtilisin/kexin type 9 (PCSK9), which inhibits ABCA1 and ABCG1, further promoting lipid accumulation in macrophages [128]. Inhibition of PCSK9 with SBC-115076 alleviates lipid accumulation and reduces atherosclerosis severity, indicating that PCSK9 inhibition has therapeutic potential in HHcy-driven atherosclerosis [128].

HHcy also significantly affects T cell proliferation and the secretion of pro-inflammatory cytokines. HHcy promotes inflammation by upregulating IL-17A expression in T lymphocytes through an epigenetic mechanism involving RNA methylation [129]. Specifically, Hcy induces the expression of NSun2, a tRNA methyltransferase that methylates IL-17A mRNA at cytosine C466 within its coding region. Methylation enhances IL-17A translation without altering mRNA stability. NSun2-deficient models exhibited markedly reduced IL-17A levels, confirming the essential role of IL-17A in this pathway. Therefore, HHcy-induced inflammation is partially mediated by the NSun2-dependent methylation of IL-17A, which contributes to chronic inflammatory conditions [129].

Another study highlighted the role of Hcy in activating T cells by enhancing ER-mitochondria coupling, resulting in metabolic reprogramming. This interaction increases mitochondrial ROS production, calcium signaling, mitochondrial mass, and respiration [130]. Blocking mitochondrial ROS production, calcium signals, or respiration significantly diminishes Hcy-induced T cell activation, including IFN-γ secretion and cell proliferation [130]. These findings underscore the importance of ER–mitochondria interactions in Hcy-driven T cell activation and related inflammatory responses.

Moreover, research has demonstrated the involvement of PKM2 in the metabolic reprograming of CD4^+^ T cells and its role in HHcy-accelerated atherosclerosis [131]. HHcy has been shown to induce PKM2 upregulation in CD4^+^ T cells *via* the PI3K/Akt/mTOR pathway, enhancing glycolysis and lipid metabolism [131]. PKM2 deficiency in CD4^+^ T cells reduces IFN-γ secretion and inhibits HHcy-driven inflammation [131]. Additionally, PKM2 knockout was found to reduce early atherosclerotic lesions in ApoE^−/−^ mice, indicating the pivotal role of PKM2 in HHcy-mediated T cell activation and the progression of atherosclerosis [131].

Additionally, shikonin (SKN), a natural naphthoquinone, has been shown to attenuate HHcy-induced atherosclerosis in ApoE^−/−^ mice by inhibiting metabolic reprogramming in CD4^+^ T cells [132]. SKN reduces PKM2 activity, suppressing glycolysis and oxidative phosphorylation (OXPHOS) in CD4^+^ T cells, leading to decreased IFN-γ secretion and reduced pro-inflammatory macrophage polarization [132]. SKN also disrupts the glycolytic-lipogenic axis, reducing lipid accumulation in T cells and mitigating HHcy-driven atherosclerosis.

The role of S-nitrosoglutathione reductase (GSNOR) in HHcy-induced T cell activation and atherosclerosis has also been explored [133]. HHcy upregulates GSNOR, which catalyzes the denitrosylation of proteins in T cells, leading to increased secretion of inflammatory cytokines, such as IL-2 and IFN-γ, thus promoting T cell proliferation [133]. GSNOR enhances Akt phosphorylation at Ser473 by reducing its S-nitrosylation at Cys224, further driving T cell activation [133]. GSNOR knockout mice exhibit reduced T cell activation and atherosclerosis, suggesting that targeting GSNOR represents a promising therapeutic strategy for mitigating HHcy-induced CVD.

B cells, particularly those involved in antigen presentation, are significantly implicated in the acceleration of atherosclerosis by HHcy. A previous study revealed that HHcy promotes B cell–T cell interactions within the vascular adventitia, as shown by single-cell RNA sequencing (scRNA-seq) in a mouse model [95]. The authors found that HHcy enhances the abundance of antigen-presenting B cells, which upregulate major histocompatibility complex class II (MHCII)-related genes [95]. These B cells interact with CD4^+^ T cells, leading to increased immune activation, as evidenced by the increased secretion of IFN-γ and IL-2. Additionally, HHcy promotes nuclear translocation of PKM2 in B cells, which binds to the CIITA promoter, inducing MHCII expression and driving B cell-mediated T cell activation. This novel PKM2-CREB1-CIITA axis plays a critical role in HHcy-driven atherosclerosis [95].

Furthermore, another study explored the mechanism by which Hcy activates B cells through PKM2-dependent metabolic reprogramming [134]. Hcy was found to promote both glycolysis and oxidative phosphorylation in B cells, with a preference for glycolysis, to meet the energy demands of B cell proliferation and antibody secretion. This metabolic shift was shown to be mediated by the Akt-mTOR signaling pathway. Moreover, inhibition of PKM2 using shikonin reversed Hcy-induced metabolic changes, B cell activation, and the early stages of atherogenesis in a mouse model of HHcy [134].

The role of IL-37, an anti-inflammatory cytokine, in HHcy-induced inflammation has also been investigated [135]. IL-37 levels were shown to be significantly reduced in patients with HHcy in both serum and peripheral blood mononuclear cells (PBMCs) [135]. In the same study, exogenous Hcy stimulation was shown to further downregulate IL-37 expression in PBMCs, leading to increased release of pro-inflammatory cytokines such as IL-1β, IL-6, and TNF-α [135]. *In vitro* experiments using 293 T cells demonstrated that IL-37 overexpression attenuated Hcy-induced cellular injury, reduced lactate dehydrogenase release, and lowered the secretion of inflammatory cytokines [135]. These findings suggest that IL-37 protects against Hcy-induced vascular inflammation by reducing inflammatory responses and cellular damage.

Another study demonstrated that emodin inhibits Hcy-induced CRP production in VSMCs by modulating PPARγ expression and the ROS-ERK1/2/p38 signaling pathway [136]. Emodin significantly reduced both the mRNA and protein levels of CRP in a dose-dependent manner, decreased ROS production, and downregulated ERK1/2 and p38 phosphorylation [136]. These results indicate that emodin exerts anti-inflammatory and anti-atherosclerotic effects by interfering with ROS-MAPK signaling and restoring PPARγ expression.

The extract of *Chrysanthemum coronarium L.* (CC) has also been shown to mitigate Hcy-induced vascular inflammation in human aortic VSMCs [137]. Hcy promotes VSMC proliferation, migration, and phenotypic switching from a contractile to a synthetic state, processes that are critical to atherosclerosis development. CC significantly reduced these effects by lowering Hcy-induced ROS production and the expression of pro-inflammatory markers, such as Nox4, soluble epoxide hydrolase (sEH), ICAM-1, and VCAM-1 [137]. CC was also found to enhance the expression of contractile proteins, including α-SMA, calponin, and SM22α, effectively reversing Hcy-induced phenotypic changes [137]. These findings indicate that CC could serve as a therapeutic or preventive agent against Hcy-induced vascular inflammation and atherosclerosis.

The protective effects of exogenous H_2_S on HHcy-induced EC (EC) damage, platelet activation, and neutrophil extracellular trap (NET) formation have also been examined [138]. In a methionine-induced HHcy rat model, the H_2_S donor NaHS was found to mitigate HHcy-induced EC injury by reducing ROS production, platelet activation, and NET formation. Mechanistically, NaHS inhibited platelet P-selectin expression and NET formation through modulation of the phospho-p38 MAPK pathway, providing protection against thrombosis [138].

Furthermore, the traditional Chinese medicine Shexiangbaoxin (SXBX) has been shown to inhibit HHcy-induced VSMC dedifferentiation [139]. HHcy promotes the proliferation, migration, and phenotypic transformation of VSMCs through the activation of NLRP3 inflammasomes [139]. The SXBX pill suppresses these processes by inhibiting NLRP3 inflammasome activation *via* the ERK and p38 MAPK pathways. Knockdown of NLRP3 or inhibition of the ERK/p38 pathway effectively reversed the effects of HHcy on VSMC dedifferentiation, highlighting the therapeutic potential of SXBX in managing HHcy-induced atherosclerosis [139].

In conclusion, the interplay between HHcy and inflammation constitutes a pivotal axis in the pathogenesis and progression of atherosclerosis. As mounting evidence suggests, elevated Hcy levels not only intensify vascular inflammation but also drive immune dysregulation, endothelial dysfunction, and plaque instability through a range of complex molecular pathways. The activation of macrophages, T cells, and B cells, coupled with mechanisms such as oxidative stress, ER stress, and inflammasome activation, underscores the central role of HHcy in promoting the development and progression of atherosclerotic lesions.

Although substantial advances have been made in identifying the inflammatory mechanisms underlying HHcy-induced atherosclerosis, several gaps remain. Future research should clarify the specific contributions of epigenetic and post-translational modifications that connect HHcy to immune activation. Additionally, exploring the therapeutic potential of novel pathways, such as the CNTF-MAFK axis and the SMAD7-NF-κB pathway, has promise in mitigating HHcy-induced vascular inflammation. Further investigation into the interactions between Hcy metabolism, lipid homeostasis, and immune responses could yield new biomarkers for early detection and lead to more effective therapeutic strategies aimed at lowering cardiovascular risk in patients with HHcy.

### 4.2. HHcy and oxidative stress

Hcy, a sulfur-containing amino acid, is highly susceptible to autoxidation in plasma, resulting in the production of ROS. This oxidative process contributes to cellular damage and dysfunction, particularly through oxidative stress mechanisms [140]. *Ex vivo* studies have shown that HHcy impairs vascular relaxation because of increased superoxide anion (O2^−^) generation [141,142]. A study involving 685 Australian Caucasians found a significant correlation between elevated plasma Hcy levels and extracellular superoxide dismutase (EC-SOD), suggesting that high Hcy levels trigger oxidative stress, leading to compromised vascular function [143]. The adverse effects of Hcy on vascular health are largely attributable to its disruption of redox homeostasis [144].

Previous research has demonstrated that oxidative stress induced by HHcy correlates with decreased thioredoxin levels in cellular models [145]. Increased thioredoxin expression reduces ROS production associated with HHcy [146]. Wu et al. [147] reported a clinical association between elevated Hcy levels in patients with CAD and diminished thioredoxin activity, suggesting a potential link between HHcy and the exacerbation of CAD.

EC-SOD, an antioxidative enzyme with anti-atherosclerotic properties, is predominantly synthesized by VSMCs and accumulates in the ECM between vascular endothelial and smooth muscle cells. Ma et al. [148] found that HHcy promotes oxidative stress by downregulating EC-SOD expression through DNA hypomethylation mediated by DNMT1. In both ApoE-deficient mice and macrophages, decreased EC-SOD levels were shown to lead to higher O_2_^−^ concentrations, aggravating atherosclerosis. Folate and vitamin B12 supplementation mitigated these effects, suggesting that epigenetic regulation of EC-SOD is a key mechanism in Hcy-induced oxidative stress [148]. Further research by Feng et al. [149] revealed that Hcy elevates malondialdehyde (MDA) levels and increases ICAM-1 expression while suppressing SOD2 and endothelial nitric oxide synthase (eNOS) at both protein and mRNA levels [149]. These oxidative effects are linked to *SORBS1* hypermethylation, which further downregulates SOD2 expression [149]. Folate and vitamin B12 supplementation reversed these changes, reducing ICAM-1 levels and restoring SOD2 and eNOS expression, highlighting a potential therapeutic strategy for Hcy-induced atherosclerosis [149].

Novel therapeutic approaches have been explored to combat HHcy-induced oxidative damage. Ding et al. [150] developed cerium vanadate nanorods as SOD-mimetic nanozymes, which demonstrated significant antioxidant activity by scavenging ROS and preserving mitochondrial function in hyperhomocysteinemic rats. These nanorods reduced myocardial infarction and improved heart function without causing toxicity, making them a promising treatment for HHcy-related cardiovascular damage [150]. In a separate study, moxibustion, a traditional Chinese therapy, was shown to reduce serum Hcy and S-adenosylhomocysteine levels while enhancing the activities of antioxidant enzymes such as SOD and heme oxygenase-1 (HO-1) [151]. This intervention improved vascular endothelial function, suggesting its potential to prevent atherosclerosis.

Hcy has also been linked to the upregulation of CTGF in VSMCs, which may accelerate atherosclerosis. Liu et al. [92] demonstrated that Hcy upregulates CTGF expression through PKC activation and ROS generation, leading to ECM accumulation and atherosclerotic plaque destabilization [92].

Oxidative stress associated with HHcy is intricately linked to inflammation, with poly (ADP-ribose) polymerase (PARP) playing a key role. Upon activation by DNA damage, PARP consumes NAD^+^ and ATP, leading to cellular dysfunction and death [152,153]. PARP also modulates the expression of inflammatory mediators *via* the NF-κB pathway [154,155]. Studies in ApoE^−/−^ mice fed a high-methionine diet (HMD) have demonstrated that HHcy increases atherosclerotic lesion size, induces DNA damage, and activates PARP and pro-inflammatory factors [156]. Interestingly, PARP inhibition reduced lesion size by 40% in HHcy-induced atherosclerosis, despite having no significant effect in hyperlipidemic mice without elevated Hcy.

Innovative imaging techniques have been developed to study oxidative stress in Hcy-induced atherosclerosis. Tang et al. [157] designed a fluorescent nanoprobe to detect hypochlorous acid (ClO^−^) fluxes during Hcy stress, providing real-time insights into oxidative stress dynamics [157]. Folic acid and N-acetylcysteine reduced ClO^−^ levels, confirming their protective effects against Hcy-induced oxidative stress [157].

The role of NADPH oxidase in Hcy-driven oxidative stress has also been well documented [145]. Early in atherosclerosis, ECs recruit monocytes and T cells through adhesion molecules and chemokines, such as MCP-1, which directs monocyte and T-cell migration into the arterial intima *via* interaction with the CC chemokine receptor 2 (CCR2) [63]. At pathophysiological levels, Hcy modifies monocyte function by upregulating the expression and secretion of MCP-1 and IL-8. This is driven by increased intracellular ROS production, primarily *via* NADPH oxidase, and involves the calmodulin and PKC pathways. ROS generated by HHcy activate mitogen-activated protein kinases (p38 and ERK1/2) and NF-κB, which are modulated by PPARγ activators [64].

Hcy also enhances ROS production in monocytes/macrophages through NADPH oxidase [158], leading to the upregulation and translocation of redox factor-1 (Ref-1), which enhances the DNA-binding activity of NF-κB, increases the expression of MCP-1, and amplifies the inflammatory response [159].

Gao et al. [160] demonstrated that Herpud1 deficiency alleviates Hcy-induced atherosclerosis by reducing oxidative stress and inflammation through suppression of the JNK/AP1 pathway. The same study also found that Herpud1 knockout decreased amyloid-β40 (Aβ40) expression, which is implicated in both Alzheimer’s disease and atherosclerosis, and improved endothelial function by promoting proliferation and reducing apoptosis, suggesting that Herpud1 represents a therapeutic target in Hcy-induced atherosclerosis [160].

Cheng et al. [161] showed that the glucagon-like peptide-1 (GLP-1) analog exendin-4 alleviates Hcy-induced endothelial dysfunction by reducing oxidative and ER stress. Exendin-4 activates the AMPK/ERO1α pathway, improving protein folding and lowering ROS production, thereby restoring endothelial function in both *in vitro* and *in vivo* models of HHcy [161]. These findings suggest that GLP-1 analogs could serve as a therapeutic option for CVDs linked to Hcy-induced endothelial damage [161].

Recent research has demonstrated the protective effects of Picroside II (P-II) against HHcy-induced endothelial damage [162]. HHcy increases oxidative stress, inflammation, and apoptosis by upregulating lectin-like oxidized low-density lipoprotein receptor-1 (LOX-1) and downregulating sirtuin-1 (SIRT1). P-II alleviates these effects by enhancing SIRT1 expression and reducing LOX-1 levels, thereby decreasing ROS production, NADPH oxidase activity, and NF-κB activation [162]. These findings suggest that P-II mitigates endothelial injury through the SIRT1/LOX-1 signaling pathway.

Sodium tanshinone IIA sulfonate (STS) has demonstrated protective effects against Hcy-induced endothelial injury by reducing oxidative stress, mitochondrial dysfunction, and apoptosis. STS activates the AKT/MAPK and SIRT1/NRF2/HO-1 pathways, whereas the NNMT/MNA pathway enhances its vascular protective effects [163]. Similarly, Ji et al. [164] demonstrated that physcion, a tetra-substituted anthraquinone, prevents Hcy-induced endothelial dysfunction by activating the Ca^2+^ and Akt-eNOS-NO signaling pathways. Physcion has been shown to ameliorate oxidative stress, mitochondrial dysfunction, and apoptosis in both *in vitro* and *in vivo* models of HHcy by restoring NO production and inhibiting the MAPK pathway, making it a potential therapeutic agent for CVDs associated with HHcy [164].

In another study, Hu et al. [165] examined the effects of catalpol, an iridoid glucoside from Rehmannia glutinosa, on HHcy-induced oxidative stress, inflammation, and apoptosis in human aortic endothelial cells. Catalpol was shown to significantly inhibit HHcy-induced injuries by reducing ROS production, improving antioxidant levels, and decreasing lipid peroxidation [165]. The protective effects of catalpol were mediated through the inhibition of the Nox4/NF-κB and GRP78/PERK pathways, which are critical regulators of oxidative and ER stress, respectively [165]. These results indicate that catalpol is a potential therapeutic agent for the treatment of HHcy-related CVDs.

Pirfenidone (PFD) has been shown to reduce macrophage infiltration and oxidative stress in the iliac arteries of hyperhomocysteinemic rabbits. By restoring antioxidant enzyme activity and reducing neointimal hyperplasia, PFD may alleviate vascular inflammation and oxidative damage, making it a potential treatment for HHcy-related atherosclerosis [166].

Li et al. [167] demonstrated that probucol effectively decreases Hcy-stimulated CRP production in rat aortic smooth muscle cells by regulating the HO-1/NADPH oxidase/ROS/p38 signaling pathway. The authors showed that probucol reduced oxidative stress, restored HO-1 expression and activity, and inhibited p38 phosphorylation [167]. These findings suggest that probucol may exert anti-inflammatory and anti-atherosclerotic effects by mitigating Hcy-induced vascular inflammation.

In conclusion, the complex relationship between HHcy and oxidative stress plays a crucial role in the pathogenesis of CVD, primarily through redox imbalance and inflammation. Oxidative stress from elevated Hcy levels impairs endothelial function and activates molecular pathways, including NADPH oxidase, NF-κB, and PARP, leading to vascular inflammation, apoptosis, and atherosclerotic plaque destabilization. Therapeutic approaches, such as antioxidant treatments, epigenetic modulation, and novel agents such as nanozymes, GLP-1 analogs, and traditional herbal compounds, have the potential to reduce HHcy-related oxidative damage. Future research should refine these therapies and explore the molecular targets to develop more effective and personalized treatments for HHcy-related conditions.

### 4.3. HHcy and epigenetics

Driven by the capacity to adapt to various internal and external stimuli, epigenetics remains a dynamic and rapidly advancing field in biomedical research. These adaptations modify gene expression through mechanisms such as DNA methylation, histone acetylation, and the regulatory actions of noncoding RNAs, with microRNAs (miRNAs) playing a particularly significant role. Epigenetic remodeling is fundamental to cellular biology and influences both normal physiology and the development of pathological conditions. In atherosclerosis, epigenetic changes are essential for the regulation of gene expression, with alterations in DNA and histones recognized as critical components. The role of miRNAs in atherosclerosis, especially in the context of HHcy, represents a promising avenue of research [168].

Notably, Whalen et al. [169] demonstrated that mild HHcy alone may not be sufficient to induce vascular hypomethylation or promote atherosclerosis, indicating that higher accumulations of plasma tHcy exhibit vascular toxicity and promote specific epigenetic dysregulation. Additionally, Gurda et al. [170] demonstrated that Hcy metabolites, specifically Hcy-thiolactone and N-Hcy-protein, induce distinct gene expression changes in human vascular ECs. Hcy-thiolactone primarily affects genes involved in chromatin organization, one-carbon metabolism, and lipid processes, whereas N-Hcy-protein influences genes related to cell differentiation and lipid metabolism [170]. Both metabolites play a role in modulating endothelial function, highlighting their involvement in HHcy-induced vascular dysfunction [170].

Emerging evidence suggests that the pathogenic role of HHcy in atherosclerosis may be closely linked to epigenetic changes involving miRNAs. These miRNAs could serve as biomarkers of atheroma plaque formation and progression in individuals with elevated Hcy levels. Liu and colleagues analyzed the expression of six miRNAs (miR-145, miR-155, miR-222, miR-133, miR-217, and miR-30) in hyperhomocysteinemic patients, and found that miR-217 and miR-133 were significantly upregulated in atherosclerosis, whereas miR-145, miR-155, and miR-222 were downregulated [171]. These miRNAs were correlated with lipid parameters and Hcy levels, with miR-217 showing particular promise as an early biomarker for atherosclerosis. In a separate study, Liu et al. [172] reported that miR-143 and miR-145 were significantly downregulated in hyperhomocysteinemic patients with carotid artery atherosclerosis. These miRNAs were negatively correlated with Hcy, total cholesterol, LDL, and TGs further supporting their potential as noninvasive biomarkers for predicting atherosclerosis development [172]. Both studies emphasize the need for large-scale research to validate miRNAs as reliable biomarkers of atherosclerosis in patients with HHcy, offering new diagnostic and therapeutic possibilities.

**Figure 4.**
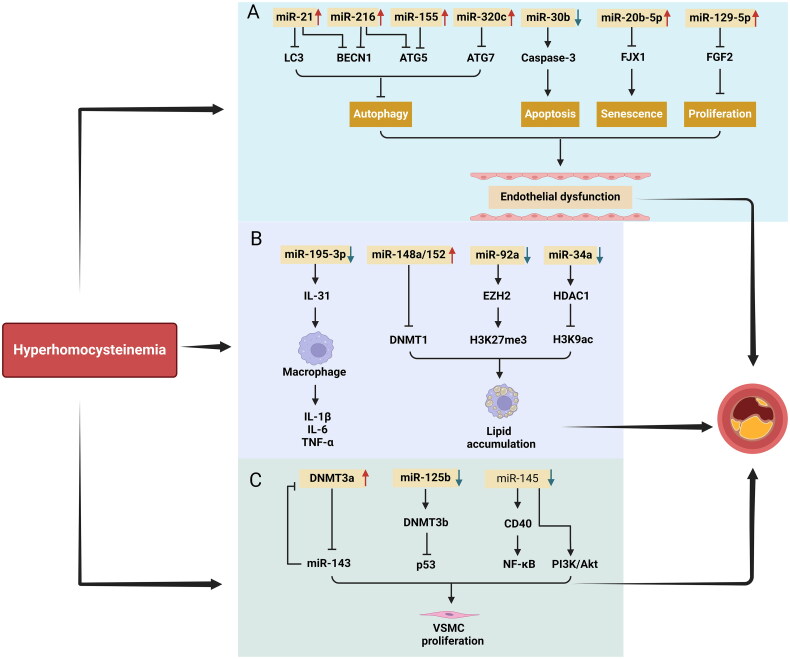
MicroRNA Dysregulation in hyperhomocysteinemia-induced atherosclerosis. Schematic illustrating the role of dysregulated microRNAs (miRNAs) in hyperhomocysteinemia (HHcy)-induced atherosclerosis. (A) In endothelial cells, hyperhomocysteinemia (HHcy) modulates microRNA-21 (miR-21), miR-216, miR-155, and miR-320c, inhibiting autophagy by targeting key autophagy-related proteins. Additionally, HHcy-induced apoptosis and senescence are regulated by miR-30b and miR-20b-5p, whereas miR-129-5p suppresses cell proliferation *via* fibroblast growth factor 2 (FGF2) downregulation. (B) In macrophages, downregulation of miR-195-3p, miR-92a, and miR-34a and upregulation of miR-148a/152 drive inflammation and lipid accumulation, contributing to atherosclerotic plaque formation. (C) Vascular smooth muscle cell (VSMC) proliferation is influenced by the miR-143/DNA methyltransferase 3 alpha (DNMT3a) and miR-125b/DNMT3b/p53 pathways. Additionally, miR-145-mediated regulation of phosphoinositide 3-kinase/protein kinase B (PI3K/Akt) signaling and CD40 expression promotes plaque development in HHcy conditions. *This figure was created using Biorender.com.*

Recent studies have also elucidated the complex role of HHcy in atherosclerosis, emphasizing the importance of microRNA dysregulation (Figure 4). These alterations significantly impact vascular function and influence smooth muscle cell proliferation, endothelial apoptosis, and macrophage inflammation, highlighting potential therapeutic targets for HHcy-related vascular dysfunction.

Witucki et al. [173] investigated how Hcy and its metabolites, Hcy-thiolactone and N-Hcy-protein, inhibit autophagy in human vascular ECs by modulating key microRNAs. Their study showed that these metabolites downregulate essential autophagy-related proteins, such as BECN1, ATG5, and ATG7, and decrease the LC3-II/LC3-I ratio, a marker of autophagy flux [173]. This inhibition was mediated by the upregulation of miR-21, miR-155, miR-216, and miR-320c, which target autophagy-related genes. Inhibition of these miRs restored autophagy, suggesting that these miRs are crucial in the endothelial dysfunction associated with HHcy.

Further research has examined the role of miR-30b in Hcy-induced apoptosis in human coronary artery ECs [174]. The findings revealed that Hcy induces apoptosis by upregulating caspase-3 expression in a concentration-dependent manner, with miR-30b being significantly downregulated during this process [174]. Overexpression of miR-30b was found to inhibit Hcy-induced apoptosis by suppressing caspase-3 expression, indicating its key role in regulating the apoptotic pathway triggered by Hcy and its potential as a therapeutic target for CVDs involving endothelial dysfunction [174].

Studies on miR-20b-5p have highlighted its role in vascular aging induced by HHcy [175]. Through RNA sequencing, researchers identified significant changes in gene expression in the arteries of HHcy model mice. miR-20b-5p was shown to directly target FJX1, and its inhibition upregulated FJX1 expression in ECs, reducing Hcy-induced oxidative stress, ROS accumulation, and cellular senescence [175]. This suggests that miR-20b-5p is a promising target for addressing HHcy-related vascular aging.

Additional investigation into the miR-129-5p/FGF2 axis revealed that Hcy inhibited FGF2 expression in HUVECs, leading to impaired cell proliferation [176]. miR-129-5p negatively regulates FGF2 expression by binding to its 3′-UTR. Knockdown of miR-129-5p upregulated FGF2 and restored HUVEC proliferation although Hcy exposure reversed these effects [176]. This indicates that the miR-129-5p/FGF2 axis plays a central role in Hcy-induced endothelial dysfunction, providing valuable insights into potential therapeutic strategies against atherosclerosis.

Recent studies have emphasized the critical role of miRNAs in regulating macrophage-driven inflammation, offering promising therapeutic strategies for addressing HHcy-induced atherosclerosis.

Xiong et al. investigated the role of miR-195-3p in alleviating Hcy-mediated atherosclerosis by targeting IL-31 [177]. Their findings demonstrated that elevated Hcy levels suppress miR-195-3p expression through DNA hypermethylation and H3K9 deacetylation, leading to increased IL-31 expression [177]. This, in turn, triggers macrophage inflammation *via* the secretion of pro-inflammatory cytokines, such as IL-1β, IL-6, and TNF-α, thus promoting atherosclerosis. Restoration of miR-195-3p expression reduced IL-31 expression, curbing inflammation and atherosclerotic plaque formation [177].

Another investigation explored the reciprocal regulation of miR-148a/152 and DNMT1 in HHcy-accelerated atherosclerosis [178]. The authors found that HHcy increased miR-148a/152 expression and decreased DNMT1 expression in ApoE-deficient mice and foam cells. miR-148a/152 directly targets DNMT1, leading to hypomethylation and lipid accumulation in foam cells [178]. Conversely, DNMT1 overexpression reduces miR-148a/152 expression by enhancing promoter methylation, thereby mitigating lipid buildup. This feedback loop is crucial in the progression of atherosclerosis driven by HHcy [178]. Further research revealed that HHcy increases EZH2 expression, leading to elevated H3K27me3 levels and subsequent lipid buildup in foam cells [179]. Overexpression of miR-92a counteracted these effects by downregulating EZH2, reducing atherosclerotic lesions, and highlighting the therapeutic potential of modulating the miR-92a/EZH2 axis [179].

Zhao et al. [180] investigated the regulatory role of miR-34a on histone deacetylase 1 (HDAC1) and its effect on Hcy-induced lipid accumulation in foam cells. They demonstrated that Hcy upregulates HDAC1, leading to decreased histone H3 acetylation at lysine 9 (H3K9ac) and promoting lipid accumulation [180]. miR-34a directly targets HDAC1, reducing its expression, which restores H3K9 acetylation and inhibits lipid accumulation [180].

Recent research has identified several critical epigenetic mechanisms through which HHcy promotes inflammation and vascular dysfunction. A key study examined the relationship between Hcy and IL-17A expression in T lymphocytes, highlighting the role of the RNA methyltransferase NSun2 [129]. It was shown that Hcy upregulates NSun2, which methylates IL-17A mRNA at cytosine 466, enhancing its translation without affecting its mRNA levels or stability [129]. This methylation increases IL-17A protein production, contributing to the pro-inflammatory response associated with HHcy [129]. In NSun2-deficient rats, Hcy-induced IL-17A upregulation was significantly reduced, confirming the essential role of NSun2 in this pathway [129]. These findings underscore the importance of NSun2-mediated methylation in HHcy-induced inflammation.

In the context of atherosclerosis, HHcy promotes the proliferation of VSMCs, a key factor in atheromatous plaque formation. Zhang et al. [181] explored the role of miR-143 in Hcy-induced VSMC proliferation. They demonstrated that Hcy downregulates miR-143 expression *via* DNA hypermethylation mediated by DNMT3a upregulation [181]. miR-143 directly targets DNMT3a, establishing a regulatory loop in which DNMT3a promotes miR-143 hypermethylation, leading to increased VSMC proliferation [181]. This miR-143/DNMT3a axis is critical in Hcy-induced atherosclerosis and provides valuable insights into therapeutic strategies targeting VSMC proliferation.

Further research explored how Hcy-induced downregulation of mitofusin-2 (MFN2) facilitates VSMC proliferation [182]. It has been shown that Hcy promotes DNA hypermethylation of the MFN2 promoter through c-Myc, which upregulates DNMT1 [182]. This hypermethylation suppresses MFN2 expression, leading to excessive cell proliferation [182]. The c-Myc/DNMT1/MFN2 pathway underscores a novel regulatory mechanism of Hcy-driven vascular dysfunction. Another key mechanism involves the miR-125b/DNMT3b/p53 axis in VSMCs [183]. Hcy-induced downregulation of miR-125b results in increased DNMT3b expression and hypermethylation of p53, leading to p53 suppression and unchecked VSMC proliferation [183]. Overexpression of miR-125b restored p53 expression, inhibiting the proliferative effects of Hcy, thereby providing insights into potential therapeutic approaches for managing atherosclerosis [183].

Research into the role of miR-217 in VSMC function revealed that Hcy activates N-methyl-D-aspartic acid receptors (NMDARs), leading to increased ROS production and activation of the CREB-PGC-1α signaling pathway [184]. miR-217 was found to inhibit NMDAR expression, reduce ROS levels, and attenuate VSMC proliferation and migration, making miR-217 a potential therapeutic target for preventing Hcy-induced vascular dysfunction [184].

Natural compounds, such as Tanshinone IIA (Tan IIA), have also been studied for their ability to counteract Hcy-induced VSMC proliferation [185]. Tan IIA modulates the miR-145/CD40 signaling pathway by restoring miR-145 expression and decreasing CD40 expression, effectively inhibiting VSMC growth [185]. The findings of this study suggest that Tan IIA could serve as a valuable therapeutic agent by regulating miRNA-related pathways in vascular disease [185].

Another protective compound, Lycium barbarum polysaccharide (LBP), reduces Hcy-induced VSMC proliferation and phenotypic transformation [186]. LBP inhibits the transition from a contractile to synthetic VSMC phenotype by suppressing the PI3K/Akt pathway and upregulating miR-145 expression [186]. This suggests that LBP has therapeutic potential in the management of atherosclerosis by targeting the VSMC dysfunction associated with HHcy.

Recent research has revealed novel epigenetic mechanisms involved in atherosclerosis linked to HHcy. Leukocyte telomere length (LTL) has emerged as a significant biomarker of aging because of the ease in sampling leukocytes. Many clinical studies have demonstrated that reductions in LTL, adjusted for age and sex, are associated with atherosclerosis and risk factors, such as elevated Hcy levels [187]. In patients with atherosclerosis, LTL levels are decreased and negatively correlated with tHcy levels [188,189]. The human telomerase reverse transcriptase (hTERT) gene, which encodes the catalytic component of telomerase, plays a crucial role in maintaining telomere length by regulating telomerase activity [188]. Hcy promotes DNA demethylation of the hTERT promoter, leading to reduced hTERT mRNA expression and telomere shortening in patients with atherosclerosis. In mouse models of Hcy-induced HHcy, the Hcy telomere accelerates attrition through similar epigenetic mechanisms [190].

Additionally, ECs exposed to chronic HHcy upregulate cellular senescence markers, including p16, p21, and p53. Hcy-induced reductions in hTERT expression occur through the promotion of CCCTC-binding factor (CTCF) binding to the demethylated hTERT promoter while inhibiting SP1 binding [190]. This reduction in telomerase activity accelerates EC aging by shortening telomeres [190]. However, supplementation with folic acid or SAM can prevent Hcy-induced hTERT promoter demethylation, restore telomerase activity, and mitigate EC senescence [190]. These findings reveal a novel epigenetic mechanism through which Hcy contributes to cardiovascular aging and the progression of atherosclerosis.

Furthermore, in patients with CI, elevated Hcy levels correlate with increased methylation of the thrombomodulin (TM) gene promoter, which results in reduced TM mRNA expression [191]. Despite this, levels of soluble TM (sTM) in the plasma are higher, likely due to endothelial damage [191]. These results suggest that Hcy-induced hypermethylation of TM contributes to CI pathogenesis by impairing the anticoagulant function of ECs [191].

The intricate relationship between HHcy and epigenetic modifications highlights the significant role of these processes in the development of atherosclerosis. HHcy influences atherosclerosis through various epigenetic mechanisms, which are primarily mediated by DNA methylation, histone modification, and miRNA dysregulation. Hcy metabolites such as Hcy-thiolactone and N-Hcy-protein contribute to chromatin reorganization and lipid metabolism alterations, further complicating vascular endothelial dysfunction. Additionally, several miRNAs, including miR-217, miR-143, and miR-125b, have been implicated in key processes, such as VSMC proliferation, endothelial apoptosis, and macrophage-driven inflammation. For instance, miR-217 and miR-195-3p are potential therapeutic targets for preventing HHcy-induced atherosclerosis.

Emerging evidence also points to the role of HHcy in telomere shortening and vascular aging through epigenetic regulation of the hTERT promoter. The link between telomere maintenance and epigenetic alterations provides further insight into the cellular senescence observed in atherosclerosis. Moreover, miR-148a/152-mediated downregulation of DNMT1 and its impact on lipid accumulation in foam cells underscores the reciprocal regulation of DNA methylation and miRNA expression during atherogenesis.

Despite these advances, further research is needed to fully delineate the specific miRNA and DNA methylation patterns involved in HHcy-driven atherosclerosis. Large-scale clinical trials are crucial for validating these epigenetic biomarkers and assessing the therapeutic potential of targeting the miRNA-DNMT axis or hTERT-related pathways. Such studies could lead to the development of epigenetically tailored interventions to mitigate the cardiovascular risks associated with elevated Hcy levels.

### 4.4. HHcy and lipoprotein metabolism

Compelling evidence suggests that HHcy significantly influences cholesterol metabolism. In a study involving 196 patients with acute myocardial infarction, Ren L et al. [107] demonstrated a direct correlation between elevated Hcy levels and increases in both total cholesterol and LDL-C. Moreover, in patients with CAD, elevated Hcy levels were associated with higher TC/HDL-C and LDL/HDL-C ratios than those with normal Hcy levels [192]. These findings indicate that Hcy may intensify lipid imbalances, particularly by elevating atherogenic cholesterol fractions.

Additionally, research focusing on patients with ACS by Wu and colleagues investigated how Hcy levels affect the efficacy of atorvastatin in modifying blood lipid profiles [193]. The study revealed that patients whose Hcy levels decreased following atorvastatin treatment experienced notable improvements in HDL-C levels and a reduction in TGs [193]. In contrast, patients with persistently high Hcy levels showed decreased HDL-C and increased TG levels despite atorvastatin therapy [193]. This suggests that the effectiveness of atorvastatin in modulating TG and HDL-C levels is influenced by Hcy levels. A reduction in Hcy levels following treatment appears to be associated with more favorable lipid profile outcomes, highlighting the potential importance of addressing HHcy in patients undergoing lipid-lowering therapy.

Elevated serum Hcy levels are strongly associated with increased total cholesterol, TG, and oxidized LDL (ox-LDL) [194]. This relationship appears to be dose-dependent, with the severity of HHcy induced by varying methionine doses directly correlating with these lipid changes. These observations suggest that HHcy contributes to atherosclerosis by influencing cholesterol metabolism. At the molecular level, studies have shown a significant increase in both the mRNA expression and enzymatic activity of 3-hydroxy-3-methylglutaryl coenzyme A reductase (HMGCR) in the livers of rats, particularly in those exposed to high concentrations of Hcy. HMGCR, a key enzyme in cholesterol biosynthesis, showed increased mRNA and protein expression in ECs treated with Hcy, further promoting cholesterol accumulation within these cells [195]. This finding provides a clear link between HHcy and disrupted cholesterol metabolism, which contributes to the pathogenesis of atherosclerosis.

HHcy also promotes the development of atherosclerosis by facilitating lipid accumulation and foam cell formation. A decrease in the expression of scavenger receptor class B type 1 (SCARB1), which mediates the uptake of cholesteryl esters from HDL in the liver, was observed in the atherosclerotic plaques of ApoE^-/-^ mice with HHcy and in foam cells exposed to Hcy. However, SCARB1 overexpression mitigated Hcy-induced lipid accumulation, highlighting the protective role of SCARB1 in cholesterol homeostasis under hyperhomocysteinemic conditions [196].

Cholesterol efflux from macrophages, a critical process for preventing foam cell formation, is mediated by ABCA1 and ABCG1. ABCA1 primarily facilitates cholesterol efflux to lipid-free apoA-I, whereas ABCG1 supports cholesterol transport to HDL particles [197,198]. However, Hcy treatment decreased the levels of both ABCA1 and ABCG1, thereby impairing cholesterol removal from macrophages and promoting foam cell formation [127].

Furthermore, Hcy has been shown to upregulate the expression of CD36, a class B scavenger receptor expressed on monocytes and macrophages that is crucial for the uptake of ox-LDL and formation of foam cells—key contributors to atherosclerotic plaque development [199]. The effect of Hcy on CD36 expression has been confirmed in studies showing elevated macrophage CD36 levels following Hcy exposure, enhancing ox-LDL uptake, and promoting foam cell formation [200]. Research by Thampi and colleagues further demonstrated that mice fed a Hcy-rich diet exhibited increased expression of scavenger receptors, particularly CD36 and LOX-1, in atherosclerotic lesions [201]. These findings indicate that dietary Hcy intensifies atherosclerosis in ApoE^-/-^ mice by increasing ox-LDL levels and upregulating scavenger receptor expression, reinforcing the role of HHcy in accelerating the development of atherosclerotic lesions.

These findings underscore the critical link between HHcy and dyslipidemia, suggesting that HHcy management may play a pivotal role in regulating lipoprotein levels and potentially enhancing the efficacy of lipid-lowering therapies. The evidence that HHcy disrupts lipoprotein metabolism, promotes lipid accumulation, and drives foam cell formation—processes central to atherosclerosis development—further establishes that HHcy is an active contributor to the pathogenesis of atherosclerosis rather than a passive marker. Recognizing its far-reaching impact on cholesterol regulation and plaque formation is essential for the development of more comprehensive treatment strategies.

Future research should focus on developing targeted therapies that mitigate the effects of Hcy on cholesterol metabolism and foam cell formation. These interventions have the potential to be translated into clinical practice, where they could enhance cardiovascular outcomes by addressing both dyslipidemia and the atherogenic effects of elevated Hcy levels.

## 5. Clinical strategies for the prevention and treatment of HHcy-induced atherosclerosis

Accumulating evidence strongly supports the causal role of Hcy in the pathogenesis of atherosclerosis, establishing it as a critical therapeutic target for reducing atherosclerosis risk [17]. Consequently, the prevention and treatment of HHcy have garnered significant attention in clinical research because of their essential role in mitigating CVD. As a result, numerous clinical trials have been conducted to explore various factors that can modulate Hcy levels, with the aim to identify effective strategies to manage and lower Hcy concentrations, thereby reducing the risk of atherosclerosis (Supplementary Table 1).

### 5.1. Targeted Nutritional therapies: the role of folate, B vitamins, and vitamin D in reducing Hcy and cardiovascular risk

Folate supplementation has consistently been shown to lower plasma Hcy levels, even in individuals without elevated Hcy [202]. Oral folic acid supplementation at doses of 0.5–5.0 mg/day reduces fasting Hcy levels by 25%–30% [203]. When combined with vitamin B12 supplementation (0.02–1 mg/day), an additional 7% reduction in Hcy levels can be achieved [203]. In contrast, vitamin B6 does not affect fasting Hcy levels but effectively reduces post-methionine load-induced HHcy [203]. Over the past decade, numerous studies have investigated the efficacy of combined administration of folic acid, vitamin B6, and vitamin B12. The doses of folic acid used in these studies varied widely, from below the recommended daily allowance (RDA) of 0.4 mg/day to higher doses of 5 mg/day or more [204].

A study by Verdoia et al. [205] involving 3,150 patients revealed a negative correlation between vitamin D levels and Hcy, indicating that lower vitamin D levels are associated with higher Hcy concentrations. Among individuals with vitamin D deficiency, elevated Hcy was linked to the severity of CAD [205]. However, no such relationship was observed in patients with optimal vitamin D levels, suggesting that adequate vitamin D supplementation may mitigate the adverse effects of elevated Hcy on coronary atherosclerosis [205]. These findings imply that maintaining sufficient vitamin D levels could protect against the harmful effects of Hcy, although further research is necessary to confirm this protective role.

The PACIFIC study, a double-blind, placebo-controlled trial, evaluated the effects of low (0.2 mg/day) and high (2.0 mg/day) doses of folic acid on Hcy levels [206]. While both doses significantly reduced Hcy, the higher dose achieved approximately one-third greater reduction [206]. This suggests that, while folic acid fortification in food helps to reduce Hcy levels across the population, higher doses may provide additional benefits for high-risk individuals.

Vermeulen et al. [207] conducted a 2-year randomized, placebo-controlled trial to assess the effects of Hcy-lowering therapy on subclinical atherosclerosis in healthy siblings of patients with premature atherothrombotic disease. Participants were randomized to receive daily folic acid (5 mg) and vitamin B6 (250 mg) or a placebo [207]. The treatment group experienced significant reductions in fasting Hcy (−49.7%) and post-methionine Hcy levels (−46.2%) [207]. Despite these reductions, the therapy did not significantly improve markers of peripheral or carotid atherosclerosis, as measured by the ankle-brachial pressure index and ultrasonographic assessments of carotid and femoral artery stenosis [207]. However, the treatment group showed a significant reduction in the incidence of abnormal exercise electrocardiograms (ECGs), with an odds ratio (OR) of 0.40 (*p* = 0.035), indicating a potential reduction in the risk of coronary events [207].

In a subsequent re-analysis of the trial, investigators evaluated the impact of Hcy-lowering therapy on ECG stress test outcomes and subclinical atherosclerosis in 158 participants, 78 of whom received vitamin supplementation and 80 received a placebo [208]. Over 2 years, the therapy significantly reduced fasting Hcy levels (−38.3%) and post-methionine Hcy levels (−30.6%) [208]. The treatment group exhibited fewer abnormal ECG stress test results, as indicated by both ST-segment displacement (OR: 0.41, *p* = 0.05) and the Athen QRS score (OR: 0.38, *p* = 0.005), which assesses ischemic depolarization abnormalities [208]. The Athen QRS score showed a stronger correlation with improved cardiac outcomes than the traditional ST segment analysis, suggesting that Hcy-lowering therapy may reduce cardiac risk in individuals with elevated Hcy levels, despite having no significant effect on peripheral arterial or carotid atherosclerosis.

Schnyder et al. conducted a series of randomized, double-blind, placebo-controlled trials to assess the effects of Hcy-lowering therapy with folic acid (1 mg/day), vitamin B12 (400 µg/day), and vitamin B6 (10 mg/day) on coronary restenosis following PCI.

In an initial study involving 205 patients, supplementation significantly reduced plasma Hcy levels (from 11.1 to 7.2 µmol/L, *p* < 0.001) compared to placebo [209]. After 6 months, the treatment group had a significantly larger minimal luminal diameter (1.72 mm vs. 1.45 mm, *p* = 0.02) and lower restenosis rate (19.6% vs. 37.6%, *p* = 0.01) [209], while the need for target lesion revascularization was reduced (10.8% vs. 22.3%, *p* = 0.047) [209]. The authors concluded that B-vitamin therapy significantly reduced coronary restenosis and revascularization rates after angioplasty.

In a subsequent trial involving 553 patients, Hcy-lowering therapy reduced the incidence of MACE (15.4% vs. 22.8%; relative risk [RR]: 0.68, *p* = 0.03), primarily driven by fewer target lesion revascularizations (9.9% vs. 16.0%; RR: 0.62, *p* = 0.03) [210]. Although trends toward reduced mortality and nonfatal myocardial infarction were observed, these outcomes did not reach statistical significance [210]. These findings suggest that Hcy-lowering therapy may improve post-PCI outcomes by reducing the need for repeat interventions.

In a post-hoc analysis of small coronary arteries (vessel size < 2.9 mm) involving 113 patients with 148 treated lesions, Hcy-lowering therapy significantly reduced the restenosis rate (15% vs. 42%, *p* = 0.001), indicating a 66% relative risk reduction [211]. For individual lesions, the restenosis rate was 11% in the treatment group and 39% in the control group (*p* = 0.0002) [211]. The therapy was particularly effective in balloon angioplasty-treated lesions, with an 82% relative risk reduction (7.3% vs. 40%, *p* = 0.0001) [211]. A nonsignificant trend toward benefit was observed for stented lesions (17% vs. 37%, *p* = 0.07) [211]. The treatment group also exhibited lower target lesion revascularization rates (11% vs. 28%, *p* = 0.041) [211]. These results indicate that Hcy-lowering therapy is particularly effective in reducing restenosis in small coronary arteries, particularly in patients undergoing balloon angioplasty.

Shidfar et al. [212] evaluated the effects of folate supplementation on Hcy and total antioxidant capacity (TAC) in hypercholesterolemic adults on lovastatin. After 8 weeks, folate supplementation (5 mg/day) significantly reduced Hcy levels and increased TAC, suggesting that folate may lower cardiovascular risk by decreasing Hcy and enhancing antioxidant capacity.

### 5.2. Mechanistic insights and clinical evidence: the impact of folic acid and B vitamins on endothelial function and vascular health

Since Hcy is an independent risk factor for CVD and contributes to endothelial dysfunction through oxidative stress and impaired NO bioavailability, lowering Hcy with folic acid and B vitamins has been shown to improve endothelial function by enhancing NO availability [213]. Folic acid, particularly in its active form 5-methyltetrahydrofolate (5-MTHF), improves vascular function through several mechanisms beyond simply reducing Hcy levels [214]. It increases endothelial NO bioavailability by preventing the oxidation of tetrahydrobiopterin (BH4), a critical cofactor of eNOS, thus improving eNOS coupling and NO production [214]. Additionally, 5-MTHF scavenges peroxynitrite radicals, protecting BH4 against oxidative degradation, further supporting eNOS activity and improving vascular health [214,215].

Chambers et al. [216] investigated the effect of B vitamin supplementation on vascular endothelial function in patients with CHD. In a randomized trial of 89 men, those who received folic acid (5 mg/day) and vitamin B12 (1 mg/day) for 8 weeks showed significant improvement in brachial artery flow-mediated dilation (FMD), which increased from 2.5% ± 3.2% to 4.0% ± 3.7% (*p* = 0.002), compared to the placebo group [216]. This improvement had a strong correlation with a reduction in plasma Hcy, particularly free Hcy, which decreased from 4.3 ± 1.2 to 3.0 ± 0.6 µmol/L (*p* < 0.001) [216]. The study concluded that B vitamin supplementation likely lowers cardiovascular risk in patients with CHD by reducing free plasma Hcy.

Doshi et al. [217] explored the effects of high-dose folic acid (5 mg/day) on endothelial function in patients with CAD. This randomized, placebo-controlled study of 33 patients found that folic acid significantly improved FMD within hours, with the effect persisting at 6 weeks [217]. Although Hcy levels did not change significantly in the short term (within 4 h), endothelial function improved regardless, suggesting that the beneficial effects of folic acid on endothelial function occur through mechanisms beyond Hcy reduction [217]. After 6 weeks, Hcy levels were lower, but the authors concluded that the endothelial improvements were largely due to folic acid’s direct effects on NO bioavailability and antioxidant activity rather than Hcy lowering [217].

Moens et al. [213] investigated high-dose folic acid (10 mg/day) in a double-blind, placebo-controlled crossover study involving patients with acute myocardial infarction. The results demonstrated significant improvements in FMD, independent of baseline Hcy levels, in both normohomocysteinemic and hyperhomocysteinemic patients [213]. This suggests that folic acid enhances endothelial function through mechanisms unrelated to Hcy reduction, such as by increasing NO bioavailability [213].

In contrast, Thambyrajah et al. [218] studied the effects of folic acid supplementation (5 mg/day) on endothelial function in 90 patients with CAD over 12 weeks. While folic acid significantly reduced plasma Hcy levels compared to placebo, no statistically significant improvement in FMD was observed, although there was a trend toward improvement in the folic acid group [218]. The authors concluded that although folic acid effectively lowers Hcy, larger trials are needed to confirm its potential benefits on endothelial function and cardiovascular outcomes.

Clinical trials assessing the impact of folic acid on Hcy levels have used various doses, from the recommended daily allowance of 0.4 mg/day to 5 mg/day. However, evidence suggests that exceeding the 0.4 mg/day threshold does not confer additional benefits to vascular function [204]. This plateau effect is thought to result from the saturation of 5-MTHF within vascular tissues, indicating that minimal doses of folic acid may be sufficient for optimal endothelial function [219]. Although plasma 5-MTHF levels increase with increased folic acid intake, intracellular 5-MTHF in the vasculature does not show a proportional increase with higher doses [219].

Despite the well-recognized negative impact of HHcy on vascular health, several studies have reported limited improvements in the elasticity of large arteries following Hcy-lowering interventions. Van Dijk et al. [220] conducted a 2-year randomized, placebo-controlled trial to assess the long-term effects of folic acid (5 mg/day) and vitamin B6 (250 mg/day) on blood pressure, endothelium-dependent vasodilation, and carotid artery stiffness in siblings of patients with premature atherothrombotic disease. Although the treatment group exhibited significant reductions in systolic (−3.7 mmHg) and diastolic (−1.9 mmHg) blood pressure compared to the placebo group, which suggests that cardiovascular benefits from Hcy-lowering therapy may be mediated through blood pressure reduction, no significant improvements were observed in endothelium-dependent vasodilation or carotid artery stiffness, indicating that large artery elasticity remained unaffected [220]. Moreover, the previously noted reduction in abnormal exercise ECGs may be partially explained by the decrease in systolic blood pressure [208].

Similarly, the B-PROOF study, another 2-year randomized, double-blind, placebo-controlled trial involving 2,919 elderly individuals (aged ≥ 65) with HHcy, evaluated the effects of daily supplementation with 500 µg vitamin B12 and 400 µg folic acid on arterial stiffness and cardiovascular outcomes [221]. Despite a significant reduction in Hcy levels (−3.6 µmol/L), the authors found no significant changes in pulse wave velocity (PWV) or carotid intima-media thickness (IMT), which are key markers of arterial stiffness and atherosclerosis [221]. Furthermore, there were no significant differences in the rates of myocardial infarction or cerebrovascular events between the intervention and placebo groups, although a nonsignificant trend toward fewer cerebrovascular events was observed in women [221]. The authors concluded that vitamin B12 and folic acid supplementation did not significantly improve arterial stiffness or reduce cardiovascular risk in this population.

In contrast, Shirodaria et al. [204] conducted a randomized, placebo-controlled study examining the effects of low-dose (400 µg/day) and high-dose (5 mg/day) folic acid on vascular function in patients with CAD. Over a 7-week period, both low- and high-dose folic acid treatments significantly improved endothelial function by enhancing NO bioavailability and reducing vascular oxidative stress [204]. These effects were attributed to the improved enzymatic coupling of eNOS facilitated by the increased availability of BH4, a critical cofactor of eNOS. However, high-dose folic acid did not provide any additional benefits over low-dose treatment [204]. Although plasma 5-MTHF levels increased in response to higher doses of folic acid, no further increase in vascular 5-MTHF was observed following high-dose treatment [204]. This suggests that low-dose folic acid, equivalent to levels found in dietary fortification, is sufficient to improve vascular function in patients with CAD, whereas higher doses offer no additional benefits [204]. This observation was explained by the saturation of the vascular wall with 5-MTHF after low-dose treatment, meaning that further increases in circulating folate levels were ineffective in further elevating vascular 5-MTHF. However, further studies are required to confirm these findings.

### 5.3. Challenges and insights from clinical trials: Efficacy and limitations of homocysteine-lowering therapies

Despite the well-established role of HHcy as a cardiovascular risk factor, multiple large-scale randomized clinical trials have failed to demonstrate the clinical benefits of Hcy-lowering interventions in patients with atherosclerosis, leading to ongoing scrutiny of the significance and efficacy of Hcy.

In the Vitamin Intervention for Stroke Prevention (VISP) trial, Toole et al. [222] aimed to assess whether high-dose vitamin supplementation (pyridoxine [B6], folic acid, and cobalamin [B12]) could reduce the risk of stroke recurrence, myocardial infarction, or mortality in individuals who experienced a non-disabling ischemic stroke. Although the high-dose group achieved a 2-µmol/L greater reduction in Hcy levels than the low-dose group, there was no significant difference in the rates of stroke, myocardial infarction, or death [222]. The recurrence rates were 9.2% in the high-dose group and 8.8% in the low-dose group. The trial concluded that B-vitamin supplementation for Hcy reduction did not significantly reduce recurrent vascular events [222]. However, the lack of a placebo group raises the possibility that the low-dose group achieved the maximum benefit, with no additional advantages from higher doses.

The Norwegian Vitamin (NORVIT) trial, led by Bønaa et al. [223], explored the effects of B-vitamin therapy on recurrent cardiovascular events in patients after acute myocardial infarction. Although Hcy levels were reduced by 27% after treatment, there was no significant reduction in recurrent myocardial infarction, strokes, or CHD-related deaths [223]. In contrast to the placebo group, the treatment group exhibited a trend toward increased adverse cardiovascular events, with a relative risk of 1.22 (*p* = 0.05), suggesting potential harm from Hcy-lowering therapy [223]. However, the NORVIT trial had several confounding factors, including most events occurring within the first year post acute myocardial infarction—a period when even statins may not provide full protection. Additionally, the concurrent initiation of Hcy-lowering therapy with other pleiotropic drugs might have obscured any minor benefits of such therapy. The study ultimately concluded against recommending B-vitamin therapy for secondary prevention after acute myocardial infarction because of potential adverse outcomes.

The Heart Outcomes Prevention Evaluation (HOPE)-2 study evaluated the impact of Hcy-lowering therapy on major cardiovascular events in patients with vascular disease or diabetes [224]. Despite a 2.4 µmol/L reduction in Hcy levels over 5 years, no significant reduction was observed in the composite endpoints of cardiovascular death, myocardial infarction, or stroke [224]. Although stroke incidence was reduced in the treatment group, there was an increase in hospitalizations for unstable angina [224]. The trial concluded that Hcy-lowering therapy with folic acid and B vitamins did not significantly reduce cardiovascular event risk and should not be recommended for this purpose. However, the study’s recruitment criteria did not ensure low baseline folate levels, and its statistical power was limited, particularly for subgroups in non-folate-fortified regions.

The Western Norway B Vitamin Intervention Trial (WENBIT), led by Løland et al. [225], investigated the effects of Hcy-lowering therapy on CAD progression in patients after PCI. Despite a 22% reduction in Hcy levels, the treatment had no significant effect on CAD progression. A post hoc analysis suggested that folic acid and vitamin B12 supplementation could increase the risk of rapid stenosis progression [225]. The authors concluded that Hcy-lowering therapy did not benefit coronary artery progression and might even promote adverse outcomes in some patients.

In the Study of the Effectiveness of Additional Reductions in Cholesterol and Homocysteine (SEARCH) trial, Armitage et al. [226] assessed the impact of Hcy-lowering therapy on major vascular events in patients with myocardial infarction. Over a follow-up period of 6.7 years, the therapy reduced Hcy levels by 3.8 µmol/L but did not reduce the incidence of major vascular events, including vascular mortality, stroke, and coronary events [226]. The study concluded that Hcy-lowering therapy with folic acid and vitamin B12 does not reduce cardiovascular risk in high-risk individuals [226]. However, the short follow-up period and the potential variability in the Hcy-lowering effects according to the stage of atherosclerosis were noted as limitations. The trial also did not determine whether individuals with severely elevated Hcy levels could benefit from extended treatment. These studies, along with findings from Albert et al. [227] and Ebbing et al. [228], suggest that B-vitamin supplementation does not significantly lower CVD risk or prevent adverse outcomes in high-risk populations.

Over the past decade, numerous clinical trials, including VISP, NORVIT, and HOPE-2, have assessed the effects of Hcy-lowering therapies—primarily using folic acid, vitamin B6, and vitamin B12—on cardiovascular risk. Although experimental and observational studies have suggested a link between HHcy and CVD, these trials have generally not demonstrated a significant reduction in cardiovascular events. Several important considerations help explain the obvious disconnect between observational findings and clinical trial outcomes.

A key observation is that although the RDA of folic acid (400 µg/day) can improve vascular function by increasing intracellular 5-methyltetrahydrofolate (5-MTHF), higher doses, such as 5 mg/day, do not provide additional benefits [219]. This result is attributed to the saturation of intracellular 5-MTHF levels, which plateaued despite higher plasma folic acid concentrations. Therefore, increasing folic acid intake beyond the RDA may not confer additional protective effects on vascular health.

In regions with sufficient dietary or fortified folate intake—such as North America, Australia, and New Zealand, which represented approximately 70% of the HOPE-2 study population—additional supplementation may not offer further benefits. In these populations, individuals already receive near-optimal folate levels; thus, folic acid supplementation does not significantly improve vascular function or reduce cardiovascular risk.

Another issue is the potentially insufficient vitamin B12 dosage, particularly in elderly patients who often experience absorption difficulties due to factors such as achlorhydria and intrinsic factor loss. In many trials, the vitamin B12 dosage may not have been adequate for correcting metabolic deficiencies, especially in elderly individuals. This could explain the modest or absent effects observed in the trials, as some subgroups (those with sufficient B12 or not receiving injections) showed minor improvements. Thus, the lack of observed benefits may be due to mistargeted supplementation strategies rather than the inherent inefficacy of Hcy-lowering vitamins.

Atherosclerosis is a slow, progressive disease that often takes decades to manifest. If Hcy plays a causal role in this process, the benefits of Hcy-lowering interventions may require longer observation periods than typical trial durations (0.5–7.3 years, with a median participant age around 60 years). The number of trials may have been too short to detect the significant long-term benefits of Hcy reduction on atherosclerosis progression.

Folic acid supplementation effectively reduces Hcy but may also have unintended effects on the methylation cycle. For instance, increased methylation of arginine residues can elevate ADMA levels, thereby impairing eNOS function [229]. Altered methylation patterns can also upregulate pro-atherogenic genes, potentially exacerbating cardiovascular conditions. Moreover, folic acid may promote cell proliferation through thymidine synthesis, which could theoretically worsen atherosclerosis by promoting the growth of atherosclerotic plaques [230].

In patients with established CAD, Hcy levels have not consistently improved and, in some cases, have appeared to increase risk. It is speculated that Hcy-induced smooth muscle cell proliferation might have a stabilizing effect on atherosclerotic plaques [231,232]. Lowering Hcy could destabilize vulnerable plaques, leading to plaque rupture, thrombosis, or accelerated progression of the lesions. This may partly explain why Hcy-lowering therapy did not yield the expected benefits in patients with advanced or unstable atherosclerotic lesions.

Participants in these trials often had multiple cardiovascular risk factors and were on several medications, such as statins. The interactions between Hcy-lowering therapy and other treatments—such as those targeting lipid levels, inflammation, and oxidative stress—may have influenced the outcomes. In some cases, lowering Hcy might have disrupted natural plaque stabilization processes, particularly in lipid-rich or necrotic lesions.

Although B-vitamin supplementation effectively reduces serum Hcy levels, its impact on tissue-bound or intracellular Hcy remains unclear. Hcy may exert its harmful effects directly within the vessel wall by altering vascular cell function or by irreversibly incorporating into cellular and extracellular matrix proteins. These pathological changes may be irreversible by simply lowering serum Hcy. The concept of ‘endothelial memory’, possibly mediated by epigenetic modifications, could explain the persistent cardiovascular risk despite reductions in serum Hcy levels [233].

Observational studies have suggested a dose–response relationship between t-Hcy and CVD risk, but most randomized controlled trials (RCTs) have focused on participants with normal or moderately elevated baseline Hcy levels [234]. To better assess the effectiveness of Hcy-lowering therapies, future large-scale trials should prioritize younger populations without CVD or exposure to mandatory folate fortification, as these groups may experience more significant benefits. Targeting individuals with intermediate-to-high-baseline HHcy would also provide a more appropriate assessment of cardiovascular outcomes.

These trials should have longer follow-up periods to evaluate the sustained impact of Hcy reduction in both the early and advanced stages of atherosclerosis. In addition, exploring whether low-dose, long-term folate supplementation can reduce cardiovascular events, particularly in underrepresented populations, is crucial for addressing existing knowledge gaps.

Given the limitations of existing approaches, alternative strategies to improve Hcy metabolism should be explored. These strategies could include enhancing the renal clearance of Hcy or increasing the intracellular 5-MTHF to optimize clinical outcomes. By improving our understanding of Hcy regulation within tissues, we can develop more effective therapies for reducing cardiovascular risk in HHcy populations.

A systematic review by Martí-Carvajal et al. [235] examined the efficacy of Hcy-lowering therapies for preventing cardiovascular events. This meta-analysis, which included 15 RCTs with > 71,000 participants, found no significant difference between Hcy-lowering interventions and placebo in preventing myocardial infarction or reducing overall mortality. However, a slight benefit was observed for stroke prevention, suggesting that although these therapies may reduce stroke risk, they do not significantly prevent other cardiovascular events or improve survival.

Another meta-analysis of 14 RCTs with 39,420 participants showed that folic acid supplementation significantly lowered Hcy levels and modestly reduced stroke risk, particularly in regions without mandatory folate fortification [236]. In areas with fortification, the effect was less pronounced, highlighting the importance of baseline folate status in the efficacy of folic acid for stroke prevention [236].

A separate meta-analysis of 6 RCTs found that folic acid significantly improved FMD, a marker of endothelial function, and reduced plasma Hcy levels [237]. Supplementation with 5 mg of folic acid for more than 4 weeks significantly enhanced endothelial function in patients with CAD, supporting the role of folic acid in improving vascular health [237].

### 5.4. Homocysteine-Lowering therapies in stroke prevention: Genetic, Nutritional, and clinical Perspectives

It is well established that there is an association between elevated Hcy levels and ischemic stroke subtypes, such as large artery atherosclerosis and small artery occlusion [238]. Vermeulen et al. [239] conducted a randomized, placebo-controlled trial to assess the impact of a combination of folic acid (5 mg/day) and vitamin B6 (250 mg/day) on cerebrovascular atherosclerosis and cerebral microangiopathy in the siblings of patients with premature atherosclerotic disease. Although the treatment significantly reduced fasting and post-methionine Hcy levels, there were no statistically significant improvements in magnetic resonance angiography or magnetic resonance imaging outcomes, suggesting a potential benefit of Hcy-lowering therapy that may require confirmation in larger trials.

In another randomized, double-blind, placebo-controlled study, Till et al. [240] examined the effects of vitamin supplementation on carotid IMT in patients at risk of cerebral ischemia. Over 1 year, supplementation with folic acid (2.5 mg/day), vitamin B6 (25 mg/day), and vitamin B12 (0.5 mg/day) significantly reduced plasma Hcy levels and carotid IMT, suggesting that vitamin supplementation may mitigate early atherosclerotic changes in high-risk cardiovascular patients.

Saposnik et al. [241] analyzed data from the HOPE-2 trial to investigate the effect of Hcy-lowering therapy on stroke risk, severity, and disability. In this study, participants with known CVD received daily supplementation with folic acid (2.5 mg), vitamin B6 (50 mg), and vitamin B12 (1 mg) or placebo for 5 years. Hcy-lowering therapy reduced the overall risk of stroke by 25%, with more pronounced benefits in younger participants, those with higher baseline Hcy levels, and individuals from regions without folic acid fortification [241]. However, the therapy did not significantly alter stroke severity or disability outcomes, suggesting that its primary benefit may be stroke prevention rather than improving outcomes after a stroke [241].

The China Stroke Primary Prevention Trial (CSPPT), led by Huo et al. [242], was a large-scale, randomized, double-blind clinical trial that evaluated the efficacy of enalapril combined with folic acid (0.8 mg/day) compared to enalapril alone in reducing the risk of first stroke in hypertensive Chinese adults. With more than 20,000 participants and a median follow-up of 4.5 years, the trial found that combined therapy significantly reduced the risk of first stroke, particularly ischemic stroke, with a 21% reduction in risk [242]. The benefits were more evident in individuals with low baseline folate levels. These findings underscore the role of folic acid supplementation in stroke prevention in populations without folate fortification.

Zhao et al. [243], who were also a part of the CSPPT, further investigated the interaction between Hcy levels, methylenetetrahydrofolate reductase (MTHFR) C677T polymorphism, and stroke risk. Folic acid supplementation reduced stroke risk by 21%, particularly in individuals with elevated Hcy levels and the CC/CT genotype [243]. However, no significant association was observed between Hcy and stroke risk in participants with the TT genotype, indicating a gene–Hcy interaction that may influence the efficacy of folic acid supplementation [243].

Huang et al. [244] performed a post-hoc analysis of the China Stroke Primary Prevention Trial (CSPPT) to assess the effects of folic acid supplementation on total homocysteine (tHcy) levels, focusing specifically on the MTHFR C677T polymorphism and serum folate status. This study, which included 16,413 participants with hypertension, found that folic acid therapy reduced tHcy levels by an average of 11% [244]. However, the reduction varied significantly based on genetic factors and folate levels [244]. Individuals with the TT genotype required higher serum folate concentrations (≥ 15 ng/mL) for optimal tHcy reduction, suggesting that personalized folic acid treatment could be more effective when tailored to both genetic and nutritional factors [244].

Further research explored the relationship between the MTHFR 677 C→T polymorphism, Hcy levels, and stroke risk across regions with differing folate intake [245]. In areas with low folate intake, such as Asia, the MTHFR variant had a more pronounced effect on Hcy levels and stroke risk, while in high-folate regions like the U.S. and Australia, where folate fortification is common, the genetic impact was minimal [245]. These results indicate that folate status influences the effect of MTHFR on Hcy levels and stroke risk, suggesting that Hcy-lowering strategies may be especially beneficial in regions with low folate intake.

Potter et al. [246] assessed the long-term effects of Hcy-lowering therapy on arterial inflammation in stroke patients. Despite a significant reduction in total plasma Hcy levels, the therapy did not influence arterial wall inflammation, as measured by fluorodeoxyglucose positron emission tomography (FDG-PET), nor did it correlate with carotid intima-media thickness or FMD [246]. This suggests that although plasma Hcy levels may reduce stroke risk, they do not necessarily alleviate arterial inflammation.

Further research, including meta-analyses of B-vitamin supplementation, showed modest reductions in stroke incidence (relative risk 0.93, 95% CI: 0.86–1.00), with the most significant benefits observed in studies lasting over 3 years or in groups without cereal folate fortification or those with chronic kidney disease [247]. These results support the role of B-vitamin supplementation in lowering stroke risk, especially in high-risk populations.

Kim et al. [248] examined serum Hcy levels and their link to all-cause and cause-specific mortality in a cohort of 221,356 adult Korean men. Their findings revealed a U-shaped curve, with both low (< 8.7 µmol/L) and high (> 13.0 µmol/L) Hcy levels associated with elevated risks of all-cause and CVD mortality. However, this association was not present among individuals taking vitamin supplements, suggesting vitamins may have a protective effect against Hcy-related mortality risks.

### 5.5. Alternative and adjunctive therapies for homocysteine reduction: Exploring novel approaches

In addition to traditional vitamin supplementation, other approaches targeting Hcy metabolism have also been explored. Liu et al. [249] investigated the effects of allicin, a compound derived from garlic, on carotid artery IMT and plasma Hcy levels in patients with CHD and HHcy. In this randomized study of 62 patients, those receiving allicin (40 mg three times daily for 12 weeks) experienced significant reductions in plasma Hcy (from 19.92 µmol/L to 13.18 µmol/L) and carotid IMT (from 1.28 mm to 1.13 mm) compared to the control group [249]. Furthermore, allicin significantly decreased total cholesterol and TGs, suggesting its potential role in preventing atherosclerosis by reducing Hcy and improving lipid metabolism [249].

Sohouli et al. [250] conducted a systematic review and meta-analysis of 20 RCTs involving 2676 participants and found that omega-3 polyunsaturated fatty acids (PUFAs) significantly reduced plasma Hcy by an average of 1.34 µmol/L. The effect was more pronounced at higher doses (≥ 3 g/day) and shorter durations (< 12 weeks) [250]. Omega-3 supplementation had a greater impact on individuals with elevated baseline Hcy, particularly those with renal or cardiovascular disease, likely through modulation of enzymes involved in Hcy metabolism [250].

Chen et al. [251] evaluated the efficacy of combining metoprolol and atorvastatin for the treatment of carotid atherosclerosis. In a retrospective study of 90 patients, combination therapy significantly reduced IMT, plaque score, and Hcy levels compared to MET monotherapy [251]. It also improved inflammatory markers, lipid profiles, and coagulation, demonstrating the superiority of MET plus ATO in managing CAS and reducing Hcy levels [251].

Shankle et al. [252] investigated the impact of CerefolinNAC^®^—a supplement combining L-methylfolate, methylcobalamin, and N-acetyl-cysteine (NAC)—on brain atrophy in Alzheimer’s patients with CVD related to HHcy. In a study of 67 participants, CerefolinNAC^®^ was shown to significantly slow the atrophy of the hippocampus, cortex, and forebrain, particularly in patients with CVD [252]. These results suggest that the Hcy-lowering properties of CerefolinNAC^®^ may delay brain atrophy, offering a potential treatment for neurodegenerative diseases linked to HHcy. Nonetheless, more research is needed to verify its effectiveness in reducing cardiovascular risk.

### 5.6. Hyperhomocysteinemia and atherosclerosis: Challenges, Emerging therapies, and future research directions

Despite significant advancements in the understanding of HHcy and its role in atherosclerosis, clinical trials evaluating Hcy-lowering interventions, such as the VISP, NORVIT, and HOPE-2 trials, have yielded disappointing results in terms of cardiovascular outcomes. The discrepancy between mechanistic evidence and trial outcomes highlights the need for more targeted and innovative approaches. Future research should focus on addressing the multifactorial nature of Hcy metabolism and its diverse effects on atherosclerosis progression by refining therapeutic strategies, identifying appropriate patient subgroups, and exploring novel treatment pathways.

The role of genetic polymorphisms, particularly the MTHFR C677T variant, in influencing Hcy metabolism is well established. Individuals with this polymorphism, particularly those with the TT genotype, exhibit reduced enzyme activity and elevated Hcy levels. Personalized therapeutic approaches that account for both genetic and nutritional factors, such as individualized folic acid therapy, may yield better results in reducing Hcy levels and preventing cardiovascular events. For example, patients with the TT genotype may require higher serum folate levels to achieve optimal Hcy reduction.

Recent studies have suggested an inverse correlation between vitamin D levels and Hcy, suggesting that vitamin D is a potential modulator of the effects of Hcy on atherosclerosis. However, establishing causality requires longitudinal studies to determine whether normalizing vitamin D levels can consistently mitigate the adverse impact of HHcy on coronary atherosclerosis. Future interventions should focus on identifying optimal vitamin D levels for cardiovascular protection, particularly in populations with vitamin D deficiency and elevated Hcy levels.

Emerging evidence indicates that the cardiovascular effects of Hcy may not be solely dependent on its concentration. Hcy contributes to endothelial dysfunction, oxidative stress, and reduced NO bioavailability, all of which are key mechanisms in atherosclerosis progression. Therapies that enhance NO bioavailability or reduce oxidative stress may offer broader cardiovascular protection than Hcy reduction alone. For instance, preventing BH4 oxidation and improving eNOS coupling could enhance endothelial function without directly targeting Hcy.

Exploring alternative therapeutic targets, such as Hcy metabolism and excretion enhancement, is promising. Strategies that modulate Hcy renal clearance or increase intracellular levels of 5-MTHF may provide novel approaches to improving vascular function. Such therapies could circumvent the plateauing effects observed with high-dose folic acid, which has limited efficacy in improving endothelial function beyond a certain threshold.

The future of HHcy treatment may extend beyond vitamin supplementation to include drugs targeting cysteine and related pathways for preventing HHcy-induced atherosclerosis. The enzyme CBS, crucial in the transsulfuration pathway, converts Hcy into cystathionine, which is then metabolized into cysteine. Cysteine plays a key role in maintaining cellular redox balance and detoxification. In HHcy, CBS mutations impair cysteine synthesis, leading to Hcy accumulation. Strategies to stabilize or restore CBS activity include using osmolyte to stabilize the enzyme, modulating its porphyrin ring structure to enhance its activity, and inhibiting proteasomal degradation of misfolded CBS proteins. These approaches boost CBS activity, increase cysteine production, and lower Hcy levels, all of which are essential for preventing HHcy-induced atherosclerosis.

NAC is a key therapeutic agent that acts as both an antioxidant and a replenisher of intracellular cysteine stores. By increasing cysteine levels, NAC supports glutathione synthesis, helping to reduce the oxidative stress linked to elevated Hcy. NAC does not directly convert Hcy into glutathione, but its ability to increase cysteine levels helps mitigate oxidative damage and improve metabolic regulation. Studies have shown that NAC effectively reduces Hcy levels, even in patients exposed to lead, making it a promising therapy for HHcy-induced atherosclerosis.

Taurine, a sulfur-containing amino acid derived from cysteine, has also shown promise in the treatment of HHcy. Taurine supplementation improves endothelial function, as measured by FMD, particularly in patients with elevated Hcy levels. The antioxidant properties of taurine and its role in modulating calcium signaling and NO bioavailability are crucial for vascular health, highlighting the importance of cysteine metabolism for cardiovascular protection.

Enzyme replacement therapy is another emerging approach targeting homocystinuria (HCU) and related HHcy. This therapy involves replacing CBS or cystathionine γ-lyase (CGL), which are enzymes that convert Hcy into cysteine. Early-stage clinical trials on CBS and CGL replacement therapies are ongoing, with future developments potentially including gene therapy *via* adeno-associated virus vectors or cDNA delivery to restore normal CBS function. These therapies directly enhance cysteine synthesis, thereby alleviating metabolic deficit in HHcy. Additionally, nonhuman enzymes, such as methionine γ-lyase (MGL), are being studied to break down methionine (a precursor of Hcy) and reduce Hcy accumulation.

Emerging non-vitamin therapies, such as allicin and omega-3 PUFAs, offer further cardiovascular benefits by modulating Hcy levels and improving lipid metabolism. These compounds, tested in large-scale trials, could broaden treatment options for managing HHcy and its role in atherosclerosis. Combination therapies targeting multiple atherogenic pathways—such as Hcy and lipid metabolism or inflammation—may also enhance clinical outcomes, particularly in high-risk populations with complex cardiovascular risks.

The inconsistent findings from large clinical trials, such as VISP, NORVIT, and HOPE-2, suggest that Hcy’s role as a cardiovascular risk factor is complex and context-dependent. Factors such as lipid levels, inflammation, and plaque stability likely modulate the effects of Hcy on atherosclerosis. It is speculated that in certain cases, lowering Hcy levels may destabilize vulnerable plaques, leading to adverse cardiovascular events. Future research should employ advanced imaging techniques and biomarkers to monitor plaque characteristics, endothelial function, and arterial stiffness in patients receiving Hcy-lowering therapy.

Moreover, the integration of advanced molecular and genetic technologies will be essential in future studies to identify biomarkers and subclinical indicators predictive of response to Hcy-lowering therapies. This approach facilitates earlier intervention in high-risk individuals, shifting the focus from treatment prevent atherosclerotic progression.

Future research should prioritize refining therapeutic strategies to target specific patient populations, exploring novel non-vitamin therapies, and elucidating the mechanistic pathways through which Hcy contributes to atherosclerosis. Addressing these challenges is essential for unlocking the full potential of Hcy-lowering interventions for the prevention and treatment of atherosclerotic CVD.

## 6. Conclusions and Perspectives

HHcy is an unequivocally recognized significant independent risk factor for atherosclerosis, profoundly impacting vascular biology. Elevated Hcy levels accelerate atherogenesis through mechanisms such as oxidative stress, endothelial dysfunction, inflammation, epigenetic changes, and lipoprotein metabolism disruption. This review highlights the diverse pathways through which HHcy exacerbates vascular damage, from established processes such as LDL oxidation and NO depletion to newer areas such as microRNA regulation and epigenetic modifications. Together, these findings underscore the complex and systemic nature of HHcy’s role in CVD.

Dietary and lifestyle interventions have been proven effective in lowering Hcy levels [253]. Folic acid-rich foods, including leafy green vegetables (e.g. spinach, broccoli, Brussels sprouts), and fortified cereals are known to reduce plasma Hcy. Vitamin B12, which is primarily found in animal products such as fish, eggs, and dairy, and vitamin B6 from sources such as meat, grains, and bananas, play pivotal roles in Hcy metabolism [254]. Deficiencies in these vitamins, particularly in individuals following a vegan or vegetarian diet, can intensify HHcy and its cardiovascular complications. Therefore, supplementation with these vitamins, especially for individuals at risk of deficiency, is crucial. Additionally, diets such as the Mediterranean diet, which is rich in fruits, vegetables, whole grains, and olive oil, have been linked to improved Hcy regulation and reduced cardiovascular risk [255]. Omega-3 fatty acids and magnesium supplementation further contribute to lowering Hcy levels and mitigating its harmful vascular effects [256,257].

In conjunction with dietary modifications, lifestyle modifications are essential for managing HHcy. Regular physical activity, smoking cessation, and reduced alcohol consumption significantly reduce Hcy levels and cardiovascular risk [258]. Since smoking and excessive alcohol consumption intensify Hcy elevation and vascular damage, lifestyle adjustments are critical components of a comprehensive management strategy for HHcy [259,260].

Despite these established approaches, translating mechanistic insights into effective therapeutic interventions remains challenging. Large clinical trials, such as VISP, NORVIT, and HOPE-2, which focused on B-vitamin supplementation to lower Hcy levels, have yielded inconclusive results. Recent meta-analyses have shown a modest but significant reduction in stroke risk associated with Hcy-lowering interventions, with no impact on CHD. These findings suggest that Hcy reduction alone may be insufficient to reduce cardiovascular risk, particularly in populations with genetic variability, different baseline Hcy levels, and co-existing conditions such as hypertension, diabetes, and dyslipidemia. Therefore, future strategies must adopt a more personalized approach that accounts for genetic factors (e.g. MTHFR polymorphisms) that influence Hcy metabolism and identify patients who are most likely to benefit from such interventions.

While B-vitamin supplementation remains a cornerstone of HHcy management, inconsistent cardiovascular outcomes suggest the need for more comprehensive therapeutic approaches. Non-vitamin therapies targeting oxidative stress and endothelial dysfunction present a promising therapeutic option. Recent research suggests that antioxidants, Nox inhibitors, and novel metabolic modulators, which go beyond traditional vitamin B supplementation, may more effectively counteract the vascular damage associated with HHcy. These therapies target specific molecular pathways, such as NADPH oxidase and PKC, which are directly involved in HHcy-induced endothelial dysfunction and inflammation.

Moreover, epigenetics and miRNA regulation are emerging as important areas in HHcy-related atherogenesis. Epigenetic modifications, including DNA methylation and histone alterations, provide a mechanistic link between elevated Hcy levels and persistent vascular injury. miRNAs such as miR-217, miR-133, and miR-143 have been implicated in regulating key processes such as VSCM proliferation, foam cell formation, and endothelial dysfunction. These molecules represent both potential biomarkers and therapeutic targets, and their integration into clinical practice could improve our ability to predict cardiovascular events and develop more personalized treatments.

In conclusion, although significant progress has been made in understanding the role of HHcy in atherosclerosis, critical gaps remain in translating these insights into clinical practice. To address these shortcomings, future strategies should combine tailored dietary and lifestyle interventions with innovative pharmacological therapies that go beyond traditional Hcy-lowering treatments. The integration of advanced diagnostic tools, such as genetic profiling, epigenetic markers, and miRNA-based diagnostics, will enable earlier detection and more precise intervention. A multifactorial and personalized approach can help us to more effectively mitigate the vascular damage caused by HHcy and reduce the global burden of atherosclerosis, ultimately improving cardiovascular outcomes in affected populations.

## Supplementary Material

Supplemental Material

## Data Availability

This study is a review article and does not involve the generation or analysis of new data. All data supporting this review are derived from previously published studies, which are fully cited in the article. As this is a synthesis of existing literature, no additional datasets are available. Further inquiries can be directed to the corresponding author. Figures were created with BioRender.com.
