# Supplementary material for: Role of hyperhomocysteinemia in atherosclerosis: from bench to bedside
Source: Ann Med. 2025 Feb 3;57(1):2457527. doi: 10.1080/07853890.2025.2457527 (PMC11792134; doi:10.1080/07853890.2025.2457527)
Supplement: Supplemental Material [file IANN_A_2457527_SM1464.docx]

**Supplementary Table 1** Interventional studies on hyperhomocysteinemia-induced atherosclerosis

| Studies | Participants (n) | Target population | Intervention | Outcome |
| --- | --- | --- | --- | --- |
| Ref.(1) | 3,150 | Patients undergoing coronary angiography | Observational study on the association between vitamin D levels and Hcy levels | Hcy levels:   - Inverse linear relationship between vitamin D and Hcy levels (r = −0.092, p < 0.001) - Higher Hcy levels observed in patients with lower vitamin D (p < 0.001)   CAD prevalence:   - Higher rate of CAD in patients with lower vitamin D and increased Hcy (81% vs. 77.7%, p = 0.13) - Significant increase in severe CAD (37.4% vs. 30.5%, p = 0.005). - Adjusted OR: for severe CAD: 1.29 (95% CI: 1.02–1.67, p = 0.04) |
| Ref.(2) | 348 | Chinese patients with acute ischemic stroke and large or small artery occlusion | Subgroup analysis based on Hcy metabolism-related vitamin (HMRV) deficiency (folic acid and vitamin B12 levels) | Hcy levels:   - Significantly higher in patients with HMRV deficiency compared to normal HMRV levels (p < 0.001)   Stroke risk:   - HHcy correlated with large artery atherosclerosis (OR: 1.126, 95% CI: 1.051–1.206, p = 0.001) and small artery occlusion (OR: 1.105, 95% CI: 1.023–1.193, p = 0.012)   Effect of vitamins:   - Vitamin B12 deficiency correlated with both large and small artery occlusion subtypes (OR: 0.992 and 0.995, p < 0.001 and p = 0.007, respectively) |
| Ref.(3) | 723 | Individuals with coronary heart disease, post-myocardial infarction, or unstable angina | - Group 1: 2.0 mg folic acid daily - Group 2: 0.2 mg folic acid daily - Group 3: Placebo | Hcy levels:   - Group 1: Reduced by 1.8 µmol/L (95% CI: 1.3–2.3, p < 0.001) - Group 2: Reduced by 1.2 µmol/L (95% CI: 0.8–1.7, p < 0.001) - Group 1 showed significantly greater reduction than Group 2 (0.6 µmol/L difference, p = 0.01)   Other outcomes:   - No significant effects on cholesterol, vitamin B12, or creatinine levels |
| Ref.(4) | 158 | Healthy siblings of patients with premature atherothrombotic disease | - Group 1: Folic acid (5 mg/d) + vitamin B6 (250 mg/d) for 2 years - Group 2: Placebo for 2 years | Hcy levels:   - Fasting Hcy decreased by 49.7% in treatment group (from 14.7 µmol/L to 7.4 µmol/L, p < 0.001) - Post-methionine Hcy decreased by 46.2% in treatment group (from 64.9 µmol/L to 34.9 µmol/L, p < 0.001)   Subclinical atherosclerosis (exercise ECG):   - Decreased abnormal ECG tests in treatment group (OR: 0.40; 95% CI: 0.17–0.93; p = 0.035)   Other outcomes:   - No significant effect on ankle-brachial pressure index, or carotid/femoral artery outcomes |
| Ref.(5) | 158 | Healthy siblings of patients with premature atherothrombotic disease | - Group 1: Folic acid (5 mg/d) + vitamin B6 (250 mg/d) for 2 years - Group 2: Placebo for 2 years | Hcy levels:   - Fasting Hcy decreased by 38.3% (95% CI: 27.0–49.6) in treatment group - Post-methionine Hcy decreased by 30.6% (95% CI: 20.7–40.5)   Electrocardiography (ECG) stress test:   - Abnormal ECG incidence reduced based on Athen QRS-score (OR: 0.28, 95% CI: 0.11–0.72, p = 0.008) - Traditional ST-segment analysis also showed reduced risk but with a wider confidence interval (OR: 0.38, 95% CI: 0.06–2.27, p = 0.29) |
| Ref.(6) | 205 | Patients who underwent percutaneous coronary angioplasty | - Group 1: Folic acid (1 mg/d) + vitamin B12 (400 µg/d) + vitamin B6 (10 mg/d) for 6 months - Group 2: Placebo | Hcy levels:   - Reduced from 11.1 μmol/L to 7.2 μmol/L in treatment group (p < 0.001)   Restenosis rates:   - Lower in treatment group (19.6% vs. 37.6%, p = 0.01) - Need for target lesion revascularization reduced (10.8% vs. 22.3%, p = 0.047)   Other outcomes:   - Major adverse cardiac events (MACEs): Trend toward fewer events in treatment group (12.7% vs. 24.5%, p = 0.055) |
| Ref.(7) | 553 | Patients after successful percutaneous coronary intervention (PCI) | - Group 1: Folic acid (1 mg/d) + vitamin B12 (400 µg/d) + vitamin B6 (10 mg/d) - Group 2: Placebo | Hcy levels:   - Reduced from 10.1 μmol/L to 7.5 μmol/L in treatment group (p < 0.001)   Clinical outcomes at 1 year:   - Major adverse events: Reduced incidence in treatment group (15.4% vs. 22.8%; RR: 0.68; 95% CI: 0.48–0.96; p = 0.03) - Target lesion revascularization: Lower in treatment group (9.9% vs. 16.0%; RR: 0.62; 95% CI: 0.40–0.97; p = 0.03) - Nonfatal myocardial infarction: No significant reduction (2.6% vs. 4.3%; p = 0.27) - Cardiac death: No significant difference (1.1% vs. 2.1%; p = 0.34) |
| Ref.(8) | 113 | Patients who underwent percutaneous coronary intervention (PCI) for narrowing in small coronary arteries (≤ 2.9 mm) | - Group 1: Folic acid (1 mg/d) + vitamin B12 (400 µg/d) + vitamin B6 (10 mg/d) for 6 months - Group 2: Placebo | Hcy levels:   - Reduced from 10.5 μmol/L to 7.2 μmol/L in treatment group (p = 0.005)   Restenosis rates:   - Lower in treatment group (15% vs. 42%, p = 0.001) - Significant reduction in restenosis for balloon angioplasty-only lesions (7.3% vs. 40%, p = 0.0001) - No significant reduction in stented lesions (17% vs. 37%, p = 0.07)   Target lesion revascularization:   - Reduced in treatment group (11% vs. 28%, p = 0.041)   Nonfatal myocardial infarction:   - No significant difference |
| Ref.(9) | 40 | Hypercholesterolemic adults undergoing lovastatin treatment | - Group 1: Folic acid (5 mg/d) + lovastatin (20 mg/d) for 8 weeks - Group 2: Placebo + lovastatin (20 mg/d) | Hcy levels:   - Group 1: Decreased from 13.35 ± 5.01 μmol/L to 8.43 ± 2.52 μmol/L (p = 0.001) - Group 2: No significant change   Total antioxidant capacity (TAC):   - Group 1: Increased from 1.54 ± 0.24 mmol/L to 1.96 ± 0.42 mmol/L (p < 0.001) - Group 2: No significant change |
| Ref.(10) | 89 | Men with coronary heart disease (CHD) | - Group 1: Folic acid (5 mg/d) + vitamin B12 (1 mg/d) for 8 weeks - Group 2: Placebo | Hcy levels:   - Total Hcy reduced from 13.0 ± 3.4 µmol/L to 9.3 ± 1.9 µmol/L (p < 0.001) - Free Hcy reduced from 4.3 ± 1.2 µmol/L to 3.0 ± 0.6 µmol/L (p < 0.001)   Vascular endothelial function (flow-mediated dilation):   - Improved from 2.5% to 4.0% in the treatment group (p = 0.002) - No significant improvement in the placebo group - Improvement in flow-mediated dilation significantly correlated with reduction in free Hcy (r = –0.26, p = 0.001) |
| Ref.(11) | 33 | Patients with coronary artery disease (CAD) | - Group 1: Folic acid (5 mg/d) for 6 weeks - Group 2: Placebo | Hcy levels:   - Total Hcy reduced from 10.6 ± 2.6 µmol/L to 8.3 ± 1.3 µmol/L (p < 0.001) - Free Hcy reduced from 2.05 ± 0.49 µmol/L to 1.46 ± 0.34 µmol/L (p < 0.001)   Endothelial function (flow-mediated dilation - FMD):   - Improved at 2 h after the first dose of folic acid (from 52.5 µm to 83 µm, p < 0.001) - Further improvement after 6 weeks (from 52.5 µm to 111 µm, p = 0.04)   Improvement in FMD did not correlate with Hcy reduction, indicating that the benefit was largely independent of Hcy lowering |
| Ref.(12) | 40 | Patients with post-acute myocardial infarction (AMI) undergoing coronary intervention | - Group 1: Folic acid (10 mg/d) for 6 weeks followed by placebo - Group 2: Placebo for 6 weeks followed by folic acid (10 mg/d) | Hcy levels:   - Group 1: Total Hcy reduced by 34% after 6 weeks of folic acid (from 15.0 ± 1.24 µmol/L to 9.87 ± 0.35 µmol/L, p < 0.001) - Group 2: Total Hcy reduced by 28% after crossover to folic acid (p < 0.001)   Endothelial function (FMD):   - Improved by 61.8% in group 1 after folic acid (3.98% to 6.44%, p < 0.001) - Group 2 showed similar FMD improvement after folic acid (p = 0.006)   FMD improvement:   - Not correlated with changes in Hcy or its subtypes (p > 0.05) |
| Ref.(13) | 90 | Patients with CAD with elevated plasma Hcy (> 11 µmol/L) | - Group 1: Folic acid (5 mg/d) for 12 weeks - Group 2: Placebo for 12 weeks | Hcy levels:   - Reduced by 24% in the folic acid group (from 11.7 ± 0.7 µmol/L to 9.3 ± 1.6 µmol/L, p < 0.001)   Endothelial function (EDD):   - Folic acid group showed a trend toward improved endothelial-dependent dilation (1.2% improvement, p = 0.07) - No significant difference in endothelial-independent dilation between groups (p = 0.18)   Other outcomes:   - No significant change in serum nitrite/nitrate or von Willebrand factor (vWF) between groups |
| Ref.(14) | 130 | Clinically healthy siblings of patients with premature atherothrombotic disease | - Group 1: Folic acid (5 mg/d) + pyridoxine (250 mg/d) for 2 years - Group 2: Placebo for 2 years | Hcy levels:   - Fasting Hcy decreased by 40.1% in the treatment group compared to placebo (p < 0.001) - Post-methionine Hcy decreased by 29.7% in the treatment group (p < 0.001)   Blood pressure:   - Systolic blood pressure reduced by 3.7 mm Hg (p = 0.02) and diastolic by 1.9 mm Hg (p = 0.04) in the treatment group   Other outcomes:   - No significant improvement in brachial artery endothelium-dependent vasodilation or carotid artery stiffness |
| Ref.(15) | 2,919 | Elderly individuals (≥ 65 years) with HHcy (12–50 μmol/L) | - Group 1: Vitamin B12 (500 μg/d) + folic acid (400 μg/d) for 2 years - Group 2: Placebo | Hcy levels:   - Decreased by 3.6 μmol/L in the intervention group (p < 0.001)   Arterial stiffness (pulse wave velocity - PWV):   - No significant effect on PWV between groups (p = 0.85)   Carotid intima-media thickness (IMT):   - No significant difference between groups (p = 0.76)   Cardiovascular events:   - No significant reduction in MI or overall cardiovascular events (p = 0.50) - Trend toward reduced cerebrovascular events in women (OR: 0.33, 95% CI: 0.15–0.71, p = 0.048) |
| Ref.(16) | 56 | Patients with CAD undergoing coronary artery bypass grafting (CABG) | - Group 1: Low-dose folic acid (400 µg/d) for 7 weeks - Group 2: High-dose folic acid (5 mg/d) for 7 weeks - Group 3: Placebo | Endothelial function (FMD):   - Improved significantly in both low- and high-dose groups compared to placebo (p < 0.05 and p < 0.001, respectively) - No significant difference between low- and high-dose groups   Vascular oxidative stress:   - Reduced superoxide production in both folic acid groups (p < 0.05 vs. placebo), no significant difference between doses   Other outcomes:   - Low-dose folic acid improved nitric oxide bioavailability and reduced vascular superoxide production - High-dose folic acid did not offer additional benefits compared to low-dose folic acid |
| Ref.(17) | 3,680 | Adults with non-disabling ischemic stroke | - Group 1: High-dose folic acid (2.5 mg/d) + vitamin B12 (0.4 mg/d) + vitamin B6 (25 mg/d) for 2 years - Group 2: Low-dose folic acid (20 µg/d) + vitamin B12 (6 µg/d) + vitamin B6 (200 µg/d) for 2 years | Hcy levels:   - High-dose group had a mean 2 µmol/L greater reduction in total Hcy than the low-dose group   Clinical outcomes:   - No significant difference in recurrent stroke rates (9.2% in high-dose vs. 8.8% in low-dose; RR: 1.0, 95% CI: 0.8-1.3, p = 0.80) - No significant difference in combined stroke, coronary heart disease (CHD) events, or death (18.0% in high-dose vs. 18.6% in low-dose; RR: 1.0, p = 0.61) - A 3 µmol/L lower Hcy level was associated with a 10% lower risk of stroke and a 26% lower risk of CHD in the low-dose group |
| Ref.(18) | 3,749 | Adults with acute myocardial infarction (MI) within 7 days prior to randomization | Duration: Median, 40 months:   - Group 1: Folic acid (0.8 mg/d) + vitamin B12 (0.4 mg/d) + vitamin B6 (40 mg/d) - Group 2: Folic acid (0.8 mg/d) + vitamin B12 (0.4 mg/d) - Group 3: Vitamin B6 (40 mg/d) - Group 4: Placebo | Hcy levels:   - Reduced by 27% in folic acid + B12 groups (from 13.0 µmol/L to 9.6 µmol/L)   Primary outcomes (composite of recurrent MI, stroke, sudden death):   - No significant reduction in folic acid + B12 group (RR: 1.08; 95% CI: 0.93–1.25; p = 0.31) - No benefit in vitamin B6 group (RR: 1.14; 95% CI: 0.98–1.32; p = 0.09) - Trend toward increased risk in combination therapy (RR: 1.22; 95% CI: 1.00–1.50; p = 0.05)   Secondary outcomes:   - Increased nonfatal MI in combination group (30% higher, p = 0.05) |
| Ref.(19) | 5,522 | Adults aged 55 years or older with vascular disease or diabetes | - Group 1: Folic acid (2.5 mg/d) + vitamin B12 (1 mg/d) + vitamin B6 (50 mg/d) for 5 years - Group 2: Placebo | Hcy levels:   - Decreased by 2.4 µmol/L in the active treatment group   Primary outcomes (composite of cardiovascular death, myocardial infarction, and stroke):   - No significant reduction in primary outcomes (RR: 0.95; 95% CI: 0.84 to 1.07; p = 0.41) - Stroke reduction (RR: 0.75; 95% CI: 0.59 to 0.97; p = 0.03) - Increased risk of hospitalization for unstable angina (RR: 1.24; 95% CI: 1.04 to 1.49; p = 0.02) |
| Ref.(20) | 923 | High-risk individuals with vascular disease or diabetes | - Group 1: Folic acid (2.5 mg/d) + vitamin B6 (50 mg/d) + vitamin B12 (1 mg/d) for 5 years - Group 2: Placebo | Hcy levels:   - tHcy levels correlated with increased carotid intima-media thickness (IMT) (r = 0.13; p < 0.001) and plaque calcification (p < 0.01) - After adjustments for age and risk factors, the correlation with IMT was no longer significant, but plaque calcification remained significantly associated with tHcy levels (p < 0.01)   Other outcomes:   - No significant association between plasma folate and carotid IMT, but lower folate levels were independently associated with increased plaque calcification (p < 0.05) |
| Ref.(21) | 348 | Patients who had undergone PCI | - Group 1: Folic acid (0.8 mg/d) + vitamin B12 (0.4 mg/d) + vitamin B6 (40 mg/d) for 10.5 months - Group 2: Folic acid (0.8 mg/d) + vitamin B12 (0.4 mg/d) for 10.5 months - Group 3: Vitamin B6 (40 mg/d) for 10.5 months - Group 4: Placebo | Hcy levels:   - Decreased by 22% in folic acid/vitamin B12 groups   Primary angiographic outcomes (minimum lumen diameter and diameter stenosis):   - No significant change in diameter stenosis or minimum lumen diameter in any treatment group   Post hoc analysis:   - Folic acid/vitamin B12 treatment associated with increased odds of rapid progression of coronary artery disease (OR: 1.84, 95% CI: 1.07 to 3.18, p = 0.03) |
| Ref.(22) | 12,064 | Survivors of MI | Duration: Median, 6.7 years   - Group 1: Folic acid (2 mg/d) + vitamin B12 (1 mg/d) - Group 2: Placebo | Hcy levels:   - Decreased by a mean of 3.8 µmol/L (28%) in the treatment group   Primary outcomes (major vascular events):   - No significant difference in major vascular events (25.5% in treatment group vs. 24.8% in placebo; RR: 1.04, 95% CI: 0.97–1.12, p = 0.28)   Stroke:   - No significant reduction in stroke (RR: 1.02, 95% CI: 0.86–1.21)   Cancer:   - No significant increase in cancer incidence (RR: 1.07, 95% CI: 0.96–1.19) |
| Ref.(23) | 5,442 | High-risk women (aged ≥ 42 years with a history of cardiovascular disease [CVD] or three or more coronary risk factors) | Group 1: Folic acid (2.5 mg/d) + vitamin B6 (50 mg/d) + vitamin B12 (1 mg/d) for 7.3 years  Group 2: Placebo for 7.3 years | Hcy levels:   - Reduced by 18.5% in the treatment group (a decrease of 2.27 µmol/L)   Primary outcomes (composite of myocardial infarction, stroke, coronary revascularization, or CVD mortality):   - No significant difference between groups (RR: 1.03; 95% CI: 0.90–1.19; p = 0.65) - No significant reduction in myocardial infarction (RR: 0.87; 95% CI: 0.63–1.22; p = 0.42), stroke (RR: 1.14; 95% CI: 0.82–1.57; p = 0.44), or CVD mortality (RR: 1.01; 95% CI: 0.76–1.35; p = 0.93) |
| Ref.(24) | 3,090 | Patients undergoing coronary angiography | Duration: Median, 38 months:   - Group 1: Folic acid (0.8 mg/d) + vitamin B12 (0.4 mg/d) + vitamin B6 (40 mg/d) - Group 2: Folic acid (0.8 mg/d) + vitamin B12 (0.4 mg/d) - Group 3: Vitamin B6 (40 mg/d) - Group 4: Placebo | Hcy levels:   - Reduced by 30% in the folic acid + vitamin B12 groups (from 10.8 to 7.6 µmol/L, p < 0.001)   Primary outcomes (composite of death, myocardial infarction, unstable angina, stroke):   - No significant reduction in the primary outcome (HR: 1.09; 95% CI: 0.90–1.32; p = 0.36 for folic acid/vitamin B12) - No significant effect of vitamin B6 (HR: 0.90; 95% CI: 0.74–1.09; p = 0.28)   Other outcomes:   - Slight increase in cancer incidence in the folic acid groups, though not statistically significant |
| Ref.(25) | 158 | Healthy siblings of patients with premature atherosclerotic disease | - Group 1: Folic acid (5 mg/d) + vitamin B6 (250 mg/d) for 2 years - Group 2: Placebo | Hcy levels:   - Fasting Hcy decreased by 38.7% (95% CI: 27.4–50.0) - Post-methionine Hcy decreased by 29.1% (95% CI: 19.2–39.0)   MRA and MRI outcomes:   - Nonsignificant improvements in cerebrovascular atherosclerosis and cerebral microangiopathy in the treatment group: - MRA score: OR: 0.48 (95% CI: 0.17–1.41; p = 0.18) - MRI score (white matter abnormalities): OR: 0.48 (95% CI: 0.14–1.60; p = 0.23) |
| Ref.(26) | 50 | Patients at risk for cerebral ischemia with carotid intima-media thickness (IMT) ≥ 1 mm | - Group 1: Folic acid (2.5 mg/d) + vitamin B6 (25 mg/d) + vitamin B12 (0.5 mg/d) for 1 year - Group 2: Placebo | Hcy levels:   - Reduced from 10.50 ± 3.93 µmol/L to 6.56 ± 1.53 µmol/L in the treatment group (p < 0.0001) - No significant change in the placebo group   Carotid IMT:   - Decreased from 1.50 ± 0.44 mm to 1.42 ± 0.48 mm in the treatment group (p = 0.034) - Increased from 1.47 ± 0.57 mm to 1.54 ± 0.71 mm in the placebo group - Significant difference in mean individual IMT changes between groups (–0.08 ± 0.17 mm in the treatment group vs. 0.07 ± 0.25 mm in the placebo group, p = 0.019) |
| Ref.(27) | 5,522 | Adults aged 55 years or older with cardiovascular disease or diabetes | - Group 1: Folic acid (2.5 mg/d) + vitamin B6 (50 mg/d) + vitamin B12 (1 mg/d) for 5 years - Group 2: Placebo | Hcy levels:   - Reduced by 2.2 µmol/L in the treatment group   Stroke risk:   - Stroke risk was significantly reduced (HR: 0.75; 95% CI: 0.59–0.97) - Nonfatal stroke reduced (HR: 0.72; 95% CI: 0.54–0.95) - No significant reduction in disabling stroke (HR: 0.64; 95% CI: 0.39–1.04) or fatal stroke (HR: 0.91; 95% CI: 0.54–1.53)   Functional status:   - No significant improvement in neurological deficits at 24 h or functional dependence at discharge |
| Ref.(28) | 20,702 | Hypertensive adults in China without prior stroke or myocardial infarction (MI) | Duration: Median, 4.5 years:   - Group 1: Enalapril (10 mg/d) + folic acid (0.8 mg/d) - Group 2: Enalapril (10 mg/d) | Primary stroke outcome:   - Risk of first stroke reduced by 21% in the enalapril–folic acid group (HR: 0.79; 95% CI: 0.68–0.93; p = 0.003)   Secondary outcomes:   - Ischemic stroke reduced (HR: 0.76; 95% CI: 0.64–0.91; p = 0.002) - No significant difference in hemorrhagic stroke (HR: 0.93; 95% CI: 0.65–1.34) - Composite cardiovascular events (stroke, MI, cardiovascular death) reduced by 20% (HR: 0.80; 95% CI: 0.69–0.92; p = 0.002) - No significant reduction in MI (HR: 1.04; 95% CI: 0.60–1.82) or all-cause death (HR: 0.94; 95% CI: 0.81–1.10) |
| Ref.(29) | 20,424 | Hypertensive adults in China without prior stroke or myocardial infarction | Duration: Median, 4.5 years:  Group 1: Enalapril (10 mg/d) + folic acid (0.8 mg/d)  Group 2: Enalapril (10 mg/d) | Hcy levels:   - In the control group, baseline Hcy associated with increased stroke risk among participants with the MTHFR CC/CT genotype (HR: 3.1; 95% CI: 1.1–9.2)   Stroke risk:   - Folic acid supplementation reduced stroke risk in the highest Hcy tertile in the CC/CT genotype (HR: 0.73; 95% CI: 0.55–0.97) - No significant stroke reduction in the TT genotype (HR: 0.44; 95% CI: 0.24–0.79) |
| Ref.(30) | 16,413 | Hypertensive adults aged 45 to 75 years without prior stroke or major cardiovascular events | Duration: Median, 4.5 years:   - Group 1: Enalapril (10 mg/d) + folic acid (0.8 mg/d) - Group 2: Enalapril (10 mg/d) | Hcy levels:   - Group 1: Mean reduction in Hcy levels of 1.61 µmol/L (11% reduction) - Hcy reduction varied by MTHFR genotype: TT genotype showed the largest reduction (–2.95 µmol/L), compared to CT (–1.30 µmol/L) and CC (–1.02 µmol/L) genotypes   Folate levels:   - Group 1: Significant increase in serum folate levels (mean increase of 16.2 ng/mL, p < 0.001) |
| Ref.(31) | 30 | Stroke patients with a history of ischemic stroke or transient ischemic attack (TIA) | Duration: Mean, 4 years:   - Group 1: Folic acid (2 mg/d) + vitamin B12 (0.5 mg/d) + vitamin B6 (25 mg/d) - Group 2: Placebo | Hcy levels:   - Reduced in the treatment group (8.4 µmol/L, 95% CI: 7.2–9.6) vs. placebo (11.6 µmol/L, 95% CI: 10.0–13.4; p = 0.002)   Arterial wall inflammation (assessed by 18F-FDG PET):   - No significant difference in arterial wall inflammation (mean arterial SUV: 2.0 ± 0.3 in treatment group vs. 2.1 ± 0.3 in placebo, p = 0.65)   Other outcomes:   - No correlation between mean arterial SUV and carotid intima-media thickness (CIMT) or flow-mediated dilation (FMD) |
| Ref.(32) | 62 | Patients with CHD patients with HHcy | Group 1: Allicin (40 mg three times daily) for 12 weeks  Group 2: Control group (no allicin) for 12 weeks | Hcy levels:   - Decreased significantly in the allicin group (from 19.92 ± 2.11 µmol/L to 13.18 ± 2.88 µmol/L, p < 0.01) - Smaller reduction in the control group (from 20.05 ± 2.15 µmol/L to 17.91 ± 2.09 µmol/L, p < 0.05)   Carotid IMT:   - Reduced significantly in the allicin group (from 1.28 ± 0.13 mm to 1.13 ± 0.10 mm, p < 0.01) - Smaller reduction in the control group (from 1.28 ± 0.08 mm to 1.23 ± 0.08 mm, p < 0.05)   Other outcomes:   - Significant reductions in total cholesterol (TC) and triglycerides (TG) in the allicin group compared to the control group |
| Ref.(33) | 90 | Patients with carotid atherosclerosis | - Group 1: Metoprolol (47.5 mg/d, increased to 95 mg/d as tolerated) for 24 weeks - Group 2: Metoprolol (same as Group 1) + Atorvastatin (20 mg/d) for 24 weeks | Hcy levels:   - Significant reduction in Hcy levels in the combination therapy group (p < 0.01)   Carotid IMT:   - IMT significantly decreased in the combination therapy group compared to monotherapy (p < 0.01)   Plaque score:   - Greater improvement in the combination therapy group (p < 0.01)   Inflammatory markers (MMP-9, hs-CRP):   - Significant reduction in both markers in the combination group compared to the monotherapy group (p < 0.05) |
| Ref.(34) | 67 | Patients with Alzheimer’s disease or cerebrovascular disease (CVD) with HHcy | Duration: 1.37 to 18.6 months:   - Group 1: CerefolinNAC® (L-methylfolate, methylcobalamin, N-acetyl-cysteine) - Group 2: No intervention (No HHcy + No CerefolinNAC) | Hcy levels:   - Reduced significantly in the treatment group (p < 0.05)   Brain atrophy rates:   - Slowed hippocampal atrophy by 4.25 times (p < 0.024) and cortical atrophy by 11.2 times (p < 0.0001) compared to the control group - Forebrain parenchymal atrophy significantly slowed only in patients with CVD (p < 0.0001) |

Abbreviations: Hcy: Homocysteine; tHcy: Total homocysteine; CAD: Coronary artery disease; OR: Odds ratio; CI: Confidence interval; HMRV: Homocysteine metabolism-related vitamin; HHcy: Hyperhomocysteinemia; µmol/L: Micromoles per liter; ECG: Electrocardiogram; MACE: Major adverse cardiac events; PCI: Percutaneous coronary intervention; RR: Relative risk; HR: Hazard ratio; TAC: Total antioxidant capacity; mmol/L: Millimoles per liter; CHD: Coronary heart disease; FMD: Flow-mediated dilation; AMI: Acute myocardial infarction; EDD: Endothelial-dependent dilation; vWF: von Willebrand factor; PWV: Pulse wave velocity; IMT: Intima-media thickness; CABG: Coronary artery bypass grafting; MI: Myocardial infarction; Plaque calcification: A condition where calcified plaques form within the arteries, often linked with atherosclerosis; CVD: Cardiovascular disease; MRA: Magnetic resonance angiography; MRI: Magnetic resonance imaging; CIMT: Carotid intima-media thickness; SUV: Standardized uptake value; 18F-FDG PET: Fluorodeoxyglucose positron emission tomography; TC: Total cholesterol; TG: Triglycerides; MMP-9: Matrix metalloproteinase-9; hs-CRP: High-sensitivity C-reactive protein.

**References**

1. Verdoia M, Nardin M, Gioscia R, Saghir Afifeh AM, Viglione F, Negro F, et al. Association between vitamin D deficiency and serum Homocysteine levels and its relationship with coronary artery disease. J Thromb Thrombolysis. 2021;52(2):523-31.

2. Wu G-H, Kong F-Z, Dong X-F, Wu D-F, Guo Q-Z, Shen A-R, et al. Association between hyperhomocysteinemia and stroke with atherosclerosis and small artery occlusion depends on homocysteine metabolism-related vitamin levels in Chinese patients with normal renal function. Metab Brain Dis. 2017;32(3):859-65.

3. Neal B, MacMahon S, Ohkubo T, Tonkin A, Wilcken D. Dose-dependent effects of folic acid on plasma homocysteine in a randomized trial conducted among 723 individuals with coronary heart disease. Eur Heart J. 2002;23(19):1509-15.

4. Vermeulen EG, Stehouwer CD, Twisk JW, van den Berg M, de Jong SC, Mackaay AJ, et al. Effect of homocysteine-lowering treatment with folic acid plus vitamin B6 on progression of subclinical atherosclerosis: a randomised, placebo-controlled trial. Lancet. 2000;355(9203):517-22.

5. Vermeulen EGJ, van Engeland MIA, Visser FC, Stehouwer CDA, Twisk JWR, van Campen CMC, et al. Effect of homocysteine-lowering vitamin treatment on electrocardiography stress tests in a randomized, placebo-controlled trial: comparison between ST-segment changes and Athen QRS-score. International Journal of Cardiology. 2004;93(2-3):323-4.

6. Schnyder G, Roffi M, Pin R, Flammer Y, Lange H, Eberli FR, et al. Decreased rate of coronary restenosis after lowering of plasma homocysteine levels. N Engl J Med. 2001;345(22):1593-600.

7. Schnyder G, Roffi M, Flammer Y, Pin R, Hess OM. Effect of homocysteine-lowering therapy with folic acid, vitamin B12, and vitamin B6 on clinical outcome after percutaneous coronary intervention: the Swiss Heart study: a randomized controlled trial. JAMA. 2002;288(8):973-9.

8. Schnyder G, Roffi M, Flammer Y, Pin R, Eberli FR, Meier B, et al. Effect of homocysteine-lowering therapy on restenosis after percutaneous coronary intervention for narrowings in small coronary arteries. Am J Cardiol. 2003;91(10):1265-9.

9. Shidfar F, Homayounfar R, Fereshtehnejad S-M, Kalani A. Effect of folate supplementation on serum homocysteine and plasma total antioxidant capacity in hypercholesterolemic adults under lovastatin treatment: a double-blind randomized controlled clinical trial. Arch Med Res. 2009;40(5):380-6.

10. Chambers JC, Ueland PM, Obeid OA, Wrigley J, Refsum H, Kooner JS. Improved vascular endothelial function after oral B vitamins: An effect mediated through reduced concentrations of free plasma homocysteine. Circulation. 2000;102(20):2479-83.

11. Doshi SN, McDowell IFW, Moat SJ, Payne N, Durrant HJ, Lewis MJ, et al. Folic acid improves endothelial function in coronary artery disease via mechanisms largely independent of homocysteine lowering. Circulation. 2002;105(1):22-6.

12. Moens AL, Claeys MJ, Wuyts FL, Goovaerts I, Van Hertbruggen E, Wendelen LC, et al. Effect of folic acid on endothelial function following acute myocardial infarction. Am J Cardiol. 2007;99(4):476-81.

13. Thambyrajah J, Landray MJ, Jones HJ, McGlynn FJ, Wheeler DC, Townend JN. A randomized double-blind placebo-controlled trial of the effect of homocysteine-lowering therapy with folic acid on endothelial function in patients with coronary artery disease. J Am Coll Cardiol. 2001;37(7):1858-63.

14. van Dijk RA, Rauwerda JA, Steyn M, Twisk JW, Stehouwer CD. Long-term homocysteine-lowering treatment with folic acid plus pyridoxine is associated with decreased blood pressure but not with improved brachial artery endothelium-dependent vasodilation or carotid artery stiffness: a 2-year, randomized, placebo-controlled trial. Arterioscler Thromb Vasc Biol. 2001;21(12):2072-9.

15. van Dijk SC, Enneman AW, Swart KMA, van Wijngaarden JP, Ham AC, Brouwer-Brolsma EM, et al. Effects of 2-year vitamin B12 and folic acid supplementation in hyperhomocysteinemic elderly on arterial stiffness and cardiovascular outcomes within the B-PROOF trial. J Hypertens. 2015;33(9).

16. Shirodaria C, Antoniades C, Lee J, Jackson CE, Robson MD, Francis JM, et al. Global improvement of vascular function and redox state with low-dose folic acid: implications for folate therapy in patients with coronary artery disease. Circulation. 2007;115(17):2262-70.

17. Toole JF, Malinow MR, Chambless LE, Spence JD, Pettigrew LC, Howard VJ, et al. Lowering homocysteine in patients with ischemic stroke to prevent recurrent stroke, myocardial infarction, and death: the Vitamin Intervention for Stroke Prevention (VISP) randomized controlled trial. JAMA. 2004;291(5):565-75.

18. Bønaa KH, Njølstad I, Ueland PM, Schirmer H, Tverdal A, Steigen T, et al. Homocysteine lowering and cardiovascular events after acute myocardial infarction. N Engl J Med. 2006;354(15):1578-88.

19. Lonn E, Yusuf S, Arnold MJ, Sheridan P, Pogue J, Micks M, et al. Homocysteine lowering with folic acid and B vitamins in vascular disease. N Engl J Med. 2006;354(15):1567-77.

20. Held C, Sumner G, Sheridan P, McQueen M, Smith S, Dagenais G, et al. Correlations between plasma homocysteine and folate concentrations and carotid atherosclerosis in high-risk individuals: baseline data from the Homocysteine and Atherosclerosis Reduction Trial (HART). Vasc Med. 2008;13(4):245-53.

21. Løland KH, Bleie O, Blix AJ, Strand E, Ueland PM, Refsum H, et al. Effect of homocysteine-lowering B vitamin treatment on angiographic progression of coronary artery disease: a Western Norway B Vitamin Intervention Trial (WENBIT) substudy. Am J Cardiol. 2010;105(11):1577-84.

22. Armitage JM, Bowman L, Clarke RJ, Wallendszus K, Bulbulia R, Rahimi K, et al. Effects of homocysteine-lowering with folic acid plus vitamin B12 vs placebo on mortality and major morbidity in myocardial infarction survivors: a randomized trial. JAMA. 2010;303(24):2486-94.

23. Albert CM, Cook NR, Gaziano JM, Zaharris E, MacFadyen J, Danielson E, et al. Effect of folic acid and B vitamins on risk of cardiovascular events and total mortality among women at high risk for cardiovascular disease: a randomized trial. JAMA. 2008;299(17):2027-36.

24. Ebbing M, Bleie Ø, Ueland PM, Nordrehaug JE, Nilsen DW, Vollset SE, et al. Mortality and cardiovascular events in patients treated with homocysteine-lowering B vitamins after coronary angiography: a randomized controlled trial. JAMA. 2008;300(7):795-804.

25. Vermeulen EGJ, Stehouwer CDA, Valk J, van der Knaap M, van den Berg M, Twisk JWR, et al. Effect of homocysteine-lowering treatment with folic acid plus vitamin B on cerebrovascular atherosclerosis and white matter abnormalities as determined by MRA and MRI: a placebo-controlled, randomized trial. Eur J Clin Invest. 2004;34(4):256-61.

26. Till U, Röhl P, Jentsch A, Till H, Müller A, Bellstedt K, et al. Decrease of carotid intima-media thickness in patients at risk to cerebral ischemia after supplementation with folic acid, Vitamins B6 and B12. Atherosclerosis. 2005;181(1):131-5.

27. Saposnik G, Ray JG, Sheridan P, McQueen M, Lonn E. Homocysteine-lowering therapy and stroke risk, severity, and disability: additional findings from the HOPE 2 trial. Stroke. 2009;40(4):1365-72.

28. Huo Y, Li J, Qin X, Huang Y, Wang X, Gottesman RF, et al. Efficacy of folic acid therapy in primary prevention of stroke among adults with hypertension in China: the CSPPT randomized clinical trial. JAMA. 2015;313(13):1325-35.

29. Zhao M, Wang X, He M, Qin X, Tang G, Huo Y, et al. Homocysteine and Stroke Risk: Modifying Effect of Methylenetetrahydrofolate Reductase C677T Polymorphism and Folic Acid Intervention. Stroke. 2017;48(5):1183-90.

30. Huang X, Qin X, Yang W, Liu L, Jiang C, Zhang X, et al. MTHFR Gene and Serum Folate Interaction on Serum Homocysteine Lowering: Prospect for Precision Folic Acid Treatment. Arterioscler Thromb Vasc Biol. 2018;38(3):679-85.

31. Potter K, Lenzo N, Eikelboom JW, Arnolda LF, Beer C, Hankey GJ. Effect of long-term homocysteine reduction with B vitamins on arterial wall inflammation assessed by fluorodeoxyglucose positron emission tomography: a randomised double-blind, placebo-controlled trial. Cerebrovasc Dis. 2009;27(3):259-65.

32. Liu D-S, Wang S-L, Li J-M, Liang E-S, Yan M-Z, Gao W. Allicin improves carotid artery intima-media thickness in coronary artery disease patients with hyperhomocysteinemia. Exp Ther Med. 2017;14(2):1722-6.

33. Chen Q, Fan L, Xu Y. Efficacy of metoprolol plus atorvastatin for carotid atherosclerosis and its influence on carotid intima-media thickness and homocysteine level. Am J Transl Res. 2022;14(8):5511-9.

34. Shankle WR, Hara J, Barrentine LW, Curole MV. CerefolinNAC Therapy of Hyperhomocysteinemia Delays Cortical and White Matter Atrophy in Alzheimer's Disease and Cerebrovascular Disease. J Alzheimers Dis. 2016;54(3):1073-84.
